# A new order, Entrophosporales, and three new *Entrophospora* species in Glomeromycota

**DOI:** 10.3389/fmicb.2022.962856

**Published:** 2022-11-29

**Authors:** Janusz Błaszkowski, Marisol Sánchez-García, Piotr Niezgoda, Szymon Zubek, Félix Fernández, Ana Vila, Mohamed N. Al-Yahya’ei, Sarah Symanczik, Paweł Milczarski, Ryszard Malinowski, Marta Cabello, Bruno Tomio Goto, Leonardo Casieri, Monika Malicka, Wojciech Bierza, Franco Magurno

**Affiliations:** ^1^Department of Environmental Management, Faculty of Environmental Management and Agriculture, West Pomeranian University of Technology in Szczecin, Szczecin, Poland; ^2^Department of Forest Mycology and Plant Pathology, Swedish University of Agricultural Sciences, Uppsala, Sweden; ^3^Faculty of Biology, Institute of Botany, Jagiellonian University, Kraków, Poland; ^4^R&D Department, Symborg SL, Murcia, Spain; ^5^Oman Animal and Plant Genetic Resources Center (Mawarid), Muscat, Oman; ^6^Zurich-Basel Plant Science Center, Institute of Botany, University of Basel, Basel, Switzerland; ^7^Department of Genetic, Plant Breeding and Biotechnology, Faculty of Environmental Management and Agriculture, West Pomeranian University of Technology in Szczecin, Szczecin, Poland; ^8^Instituto Spegazzini, Comisión de Investigaciones Científicas de La Provincia de Buenos Aires (CIC-PBA), La Plata, Argentina; ^9^Departamento de Botânica e Zoologia, Universidade Federal do Rio Grande do Norte, Campus Universitário, Natal, RN, Brazil; ^10^Mycorrhizal Applications LLC at Bio-Research and Development Growth Park, St. Louis, MO, United States; ^11^Institute of Biology, Biotechnology and Environmental Protection, Faculty of Natural Sciences, University of Silesia in Katowice, Katowice, Poland

**Keywords:** arbuscular mycorrhizal fungi, *Claroideoglomus*, four new taxa, morphology, new combinations, nuc rDNA, phylogenomic and phylogenetic taxonomy, *rpb1*

## Abstract

As a result of phylogenomic, phylogenetic, and morphological analyses of members of the genus *Claroideoglomus*, four potential new glomoid spore-producing species and *Entrophospora infrequens*, a new order, Entrophosporales, with one family, Entrophosporaceae (=Claroideoglomeraceae), was erected in the phylum Glomeromycota. The phylogenomic analyses recovered the Entrophosporales as sister to a clade formed by Diversisporales and Glomeraceae. The strongly conserved entrophosporoid morph of *E. infrequens*, provided with a newly designated epitype, was shown to represent a group of cryptic species with the potential to produce different glomoid morphs. Of the four potential new species, three enriched the Entrophosporales as new *Entrophospora* species, *E. argentinensis, E. glacialis*, and *E. furrazolae*, which originated from Argentina, Sweden, Oman, and Poland. The fourth fungus appeared to be a glomoid morph of the *E. infrequens* epitype. The physical association of the *E. infrequens* entrophosporoid and glomoid morphs was reported and illustrated here for the first time. The phylogenetic analyses, using nuc rDNA and *rpb1* concatenated sequences, confirmed the previous conclusion that the genus *Albahypha* in the family Entrophosporaceae sensu Oehl et al. is an unsupported taxon. Finally, the descriptions of the Glomerales, Entrophosporaceae, and *Entrophospora* were emended and new nomenclatural combinations were introduced.

## Introduction

The genus *Entrophospora* with the type species *E. infrequens* was erected in the family Endogonaceae based on a species originally described as *Glomus infrequens* (Hall, 1977), but later recognized to form spores differently (inside the neck of a sporiferous saccule; Ames and Schneider, 1979) than species producing glomoid spores sensu Morton and Redecker (2001). Glomoid spores arise at the tips of sporogenous hyphae as in *G. macrocarpum*, the type species of the genus *Glomus* and the phylum Glomeromycota, including arbuscular mycorrhizal fungi (AMF; Clements and Shear, 1931; Schüßler and Walker, 2010; Oehl et al., 2011a). Subsequently, *Entrophospora* was enlarged by *E*. *schenckii* (Sieverding and Toro, 1987) and *E. baltica* (Błaszkowski et al., 1998), producing two-walled spores with a thick, smooth wall 2 as in *E. infrequens*, as well as *E. colombiana* (Schenck et al., 1984), and *E. kentinensis* (Wu et al., 1995) having spores with three spore walls, of which the innermost wall 3 is relatively thin and usually ornamented (“beaded”), as in most *Acaulospora* species.

Morton and Benny (1990) transferred the entrophosporoid [name coined by Sieverding and Oehl (2006)] species mentioned above to the family Acaulosporaceae in the order Glomales. Sieverding and Oehl (2006) accommodated *E. baltica* and *E. infrequens* in a newly erected family, Entrophosporaceae, in the class Glomeromycetes; *E. colombiana* and *E. kentinensis* in a new genus, *Kuklospora*, in the Acaulosporaceae; and *E. schenckii* in *Intraspora* gen. nov. in the family Archaeosporaceae sensu Morton and Redecker (2001). Schüßler and Walker (2010) introduced Claroideoglomeraceae fam. nov. with *Claroideoglomus* gen. nov. into the Glomerales (orthographically corrected Glomales; Schüßler et al., 2001). The type species of the monogeneric Claroideoglomeraceae, *Claroideoglomus*, was *C. claroideum* (Schüßler and Walker, 2010), originally described as *Glomus claroides* (Schenck and Smith, 1982). Entrophosporaceae and *Entrophospora* were recognized as taxa of uncertain systematic position in the order Diversisporales, *Kuklospora* synonymized with *Acaulospora*, and *I. schenckii* renamed as *Archaeospora schenckii*. Kaonongbua et al. (2010), based on a phylogeny reconstructed from nuc 28S rDNA (28S) sequences, also rejected *Kuklospora* as a valid monophyletic group, therefore, integrated it into *Acaulospora*, and concluded that the mode of spore formation is not a genus-specific character.

Oehl et al. (2011b) synonymized Claroideoglomeraceae with Entrophosporaceae based on phylogenetic analyses of nuc 18S rDNA (18S) and 28S sequences of *E. infrequens*, three species of *Claroideoglomus*, other representatives of Glomerales, and members of eleven (in the 18S analyses) and nine (28S) other previously described families of Glomeromycota. The genus *Claroideoglomus* remained in Entrophosporaceae, and *Entrophospora* with *E. infrequens* became the type genus of this family. The same analyses also prompted Oehl et al. (2011b) to transfer *C. drummondii* and *C. walkeri*, originally described as *G. drummondii* and *G. walkeri* (Błaszkowski et al., 2006), to a newly erected genus, *Albahypha*, with *A. drummondii* and *A. walkeri*, and *G. viscosum* to a new genus, *Viscospora*, in the Entrophosporaceae. In addition, Oehl et al. (2011b) accommodated *E. baltica* in Sacculosporaceae fam. nov., as *S. baltica* in the Diversisporales.

Except for the introduction of the Sacculosporaceae, Redecker et al. (2013) rejected the other changes discussed above and resurrected Claroideoglomeraceae. They found that both 18S and 28S sequences of *E. infrequens* nest among sequences of *Claroideoglomus*, and the incongruence between morphological and molecular characters of these taxa could not be explained from either dataset.

The main argument for rejecting *Albahypha* was linked to analyses conducted by Krüger et al. (2012), which indicated sequences of *A. drummondii* and *A. walkeri* clustering among *Claroideoglomus* sequences. In addition, Redecker et al. (2013) underlined that the generic clade *Albahypha* in Oehl’s et al. (2011b) 28S tree was not supported and, therefore, rendered *Claroideoglomus* paraphyletic.

In Schüßler and Walker’s (2010) classification, Claroideoglomeraceae was a sister taxon of Glomeraceae in the Glomerales. Two phylogenies have recently been published suggesting other relationships of Claroideoglomeraceae within Glomeromycota sensu Redecker et al. (2013). Beaudet et al. (2018), using spore transcriptomic data for analyses of nine taxa from seven families, recovered *Claroideoglomus* as sister to *Ambispora* and *Paraglomus*, belonging to the orders Archaeosporales and Paraglomerales, respectively. Montoliu-Nerin et al. (2021), using single nuclei sequencing to obtain new genomes of 15 species belonging to seven families, indicated that Claroideoglomeraceae represents an autonomous, well-supported group, sister to a clade formed by Glomeraceae and Diversisporales.

Species of *Claroideoglomus*, Glomeraceae, and *Paraglomus* produce solely glomoid spores, while *Ambispora* species are dimorphic, forming acaulosporoid and glomoid morphs (Bills and Morton, 2015). Of members of the genera of the Diversisporales, only those of *Corymbiglomus, Desertispora, Diversispora, Sieverdingia, Redeckera*, and *Pacispora* produce glomoid spores (Oehl et al., 2011c; Błaszkowski, 2012; Symanczik et al., 2018; Błaszkowski et al., 2019). Glomoid spore-producing species of the genera mentioned above have one-walled spores, except for species of *Pacispora* and *Paraglomus*, which produce spores with two spore walls (Mello et al., 2013).

Schüßler and Walker (2010) and Oehl et al. (2011a) stated that the spores of *Claroideoglomus* species are morphologically distinguished by the composition of their spore wall and the phenotypic features of the spore wall components, as well as the characters of the spore subtending hypha at the spore base. According to Schüßler and Walker (2010), the spore wall of the members of *Claroideoglomus* contains a flexible, thin, colorless innermost layer. Oehl et al. (2011a) distinguished *Claroideoglomus* spores as having conspicuously funnel- or bill-shaped and colorless subtending hyphae regardless of whether the spore wall is colored or not. However, the above-mentioned morphological features not only characterize all *Claroideoglomus* species but also define glomoid spores of members of several other genera of Glomeromycota, for example, *Diversispora* species and *Orientoglomus emiratium*, a member of Glomeraceae (Błaszkowski et al., 2022). So, a reliable separation of *Claroideoglomus* species from other Glomeromycota species producing glomoid spores based exclusively on their morphology would be biased.

The data presented above confirm the widely accepted opinion that a more accurate identification and classification of AMF, including the Claroideoglomeraceae/Entrophosporaceae members, needs to be based mainly on reliable molecular data. Short sequences are usually the source of weak signals, resulting in insignificant support values for topologies of phylogenetic trees (Redecker et al., 2013). Even full-length 28S sequences failed to resolve closely related AM fungal species (Redecker et al., 2013).

The following hypotheses might explain the cause of the divergent conclusions about the Claroideoglomeraceae/Entrophosporaceae, as well as the phylogenies of *E. infrequens* and the two species considered as *C.*/*A. drummondii* and *C.*/*A. walkeri*. First, as suggested by Montoliu-Nerin et al. (2021), the conclusions of Beaudet et al. (2018) were biased by the low number of taxa analyzed. Second, we considered the vague phylogenies of *E. infrequens, C.*/*A. drummondii*, and *C.*/*A. walkeri* that resulted from the sequence under-sampling of the taxa and the use of sequences with too low taxonomic resolution. Therefore, we assumed that (i) genome-scale phylogenetic analyses will shed light on the phylogenetic placement of the family Claroideoglomeraceae/Entrophosporaceae and (ii) increased sampling and the use of sequences covering the 18S (partial), ITS1-5.8S-ITS2, and 28S (partial) nuc rDNA (=45S) segment concatenated with partial sequences of the largest subunit of RNA polymerase II (*rpb1*) will clarify the *E. infrequens, C.*/*A drummondii*, and *C.*/*A. walkeri* phylogenies. Sequences of each of the two loci generally separated even very closely related species (Krüger et al., 2012; Kohout et al., 2014; Stockinger et al., 2014). However, phylogenies reconstructed from concatenated sequences of the two unlinked loci (45S+*rpb1*) were more robust and revealed relationships unexposed when sequences of these two loci were analyzed separately (Błaszkowski et al., 2019, 2021a, 2015b,c; Yu et al., 2022).

We grew *E. infrequens* and four potential new glomoid spore-producing AMF species in trap and single-species cultures. The morphological characteristics of these glomoid fungi, particularly the colorless subtending hyphae of their pigmented spores, suggested that they may represent *Albahypha*/*Claroideoglomus* or *Diversispora*.

The aims of this study were (i) to determine which of the known hypotheses about the status and relationship of Claroideoglomeraceae/Entrophosporaceae is reliable, (ii) to clarify the position of *E. infrequens* in the Glomeromycota with the support of *rpb1* as an additional phylogenetic marker, (iii) to characterize in detail the morphology of the four potentially new AMF species and determine their phylogenetic positions among sequenced species of *Albahypha*/*Claroideoglomus* based on 45S and *rpb1* sequences, and (iv) to settle the conflict over the taxonomic status of *Albahypha* using previously available and newly generated morphological and molecular (45S and *rpb1*) data.

## Materials and methods

### Origin of the study material

*Entrophospora infrequens* and the potential new species, initially named *Claroideoglomus* 1–4, were characterized based on spores extracted from single-species pot cultures. These cultures were established from spores extracted from trap pot cultures inoculated with field-collected mixtures of rhizosphere soils and root fragments of the following plant species. The field host of *E. infrequens* was *Juniperus communis* L. growing in a pine forest on inland sand dunes of Kampinos National Park (52*^o^*19′ N, 20*^o^*45′ E), Poland. The field sample was collected by J. Błaszkowski on 26 July 1986.

The field hosts of *Claroideoglomus* 1 were *Deschampsia flexuosa* (L.) Trin and *Poa rigidifolia* Steud. These plants grew in a steppe community located in northern Tierra del Fuego (Mendoza et al., 2011) on a complex of moraine deposits and glacial-outwash plains dominated by grasses of *Chiliotrichum diffusum* (G. Forst.) Kuntze, *D. flexuosa, Empetrum rubrum* Vahl ex Willd., *Festuca gracillima* Hook., and *P. rigidifolia* (Collantes et al., 1999).

The regional climate is influenced by the proximity of the Atlantic Ocean (Paruelo et al., 1998). The mean air temperature is about 0°C in July (winter) and reaches 10°C in January (summer). The annual rainfall ranges from about 300 to 400 mm along a NE–SW gradient and is evenly distributed throughout the year. The field sample was collected by R. Mendoza, Museo Argentino de Ciencias Naturales (MACN-CONICET) “Bernardino Rivadavia,” Buenos Aires, Argentina, during the period from 1 to 15 March 2009.

*Claroideoglomus* 2 was harbored in the field within an initial community consisting of cryptogamic species and the following vascular plants: *F. vivipara* (L.) Sm., *Po. alpina* L., *Salix herbacea* L., *S. polaris* Wahlenb., and *Silene acaulis* (L.) Jacq. This community grew in a glacier foreland of Isfallglaciarän located in Tarfala valley, N Sweden (67°54′58 N, 18°35′ E). The average annual air temperature and the annual sum of rainfall in this site are 3.4°C and 503.3 mm, respectively (The Swedish Agency for Marine and Water Management, available at: www.smhi.se). The soil contained 19.28 and 3.17% of silt and clay, respectively, and its chemical properties were as follows: pH (in H_2_O), 6.3; P_2_O_5_ (mg/100 g), 4.63; Na (cmol/kg), 0.04; N (%), 0.00; and C (%), 0.08. The field sample was collected by Paulina Wietrzyk-Pełka and Michał Węgrzyn (Institute of Botany of the Jagiellonian University) on 23 July 2019.

The field inoculum containing *Claroideoglomus* 3 was collected under an initial community consisting of *Limonium sinuatum* (L.) Mill. This community grew in a Solonetz Gley sodic soil, which was hydromorphic, compact, and contained high levels of surface salt deposits. The community was located at Fortuna, Murcia, Spain (38°06′ N, 1°06′ W). The mean annual air temperature and the annual sum of rainfall in this site are 19.6°C and 300 mm, respectively. The soil contained 20.28 and 53% of silt and clay, respectively, and its chemical properties were as follows: pH (in H_2_0), 8.5; P_2_O_5_ (mg/100 g), 2.1, CaCO_3_ (%), 12.0; Mg_2_ (cmol/kg), 2967.04; K (cmol/kg), 2955; Na (cmol/kg), 1829.4; N (%), 0.90; and C (%), 0.08. The field sample was collected by Félix Fernández in November 2009.

*Claroideoglomus* 4 was isolated from samples collected in the Sultanate of Oman and in Poland. In Oman, *Claroideoglomus* 4 was associated with roots of *Prosopis cineraria* (L.) Druce. It grew in an undisturbed habit adjacent to a date palm (*Phoenix dactylifera* L.) plantation of an experimental station (22°14′ N, 59°10′ E) belonging to the Ministry of Agriculture, Fisheries and Water Resources of Oman. Summer temperatures exceed 48°C (Glennie and Singhvi, 2002). Based on the aridity index defined by the United Nations Environmental Program (UNEP, 2006), this site is characterized as Hyperarid (AI < 0.05), with annual rainfall not exceeding 100 mm (Fisher and Membery, 1998). The soil of this site was sandy loam with the following chemical properties: pH (in H_2_O), 8.2; contents of P (mg kg^–1^), 41.4; N (%), < 0.1; and organic matter (%), 1.5 (Al-Yahya’ei et al., 2011). The field sample was collected by Mohamed N. Al-Yahya’ei in August 2006.

The site in Poland where *Claroideoglomus* 4 was found is a field near Lubliniec in the Silesian Upland (50°37′ N, 18°43′ E) from where *Dominikia bonfanteae* was isolated (Błaszkowski et al., 2021b). Details about plant community and the physico-chemical properties of soil are available in Malicka et al. (2020). The climate of the region, where the study material was collected, is characterized by Błaszkowski et al. (2019).

Spores of the species originally described as *G. walkeri* and *C. hanlinii*, the latter of which was also included in our analyses (see below), originated from single-species cultures grown by J. Błaszkowski.

### Establishment and growth of trap and single-species cultures, extraction of spores, and staining of mycorrhizal structures

Methods used to establish trap and single-species cultures, growing conditions, and methods of spore extraction and staining of mycorrhizal structures were as those described previously (Błaszkowski et al., 2012). Five to ten spores of uniform morphology of each AMF species were used to establish single-species cultures.

### Microscopy and nomenclature

Morphological features of spores and phenotypic and histochemical characters of spore wall layers of the new species presented here were characterized based on at least 50-100 spores of each species mounted in water, lactic acid, polyvinyl alcohol/lactic acid/glycerol (PVLG, Omar et al., 1979), and a mixture of PVLG and Melzer’s reagent (1:1, v/v). The preparation of spores for study and photography was as described previously (Błaszkowski, 2012; Błaszkowski et al., 2012). The types of spore wall layers were defined by Walker (1983) and Błaszkowski (2012). Color names were from Kornerup and Wanscher (1983). The nomenclature of fungi and the authors of fungal names are from the Index Fungorum website http://www.indexfungorum.org/AuthorsOfFungalNames.htm. The term “glomerospores” was used for spores produced by AMF as proposed by Goto and Maia (2006).

Voucher specimens of the proposed new species [spores permanently mounted in PVLG and a mixture of PVLG and Melzer’s reagent (1:1, v/v) on slides] were deposited at ZT Myc (ETH Zurich, Switzerland; holotypes) and in the Laboratory of Plant Protection, Department of Shaping of Environment (LPPDSE), West Pomeranian University of Technology in Szczecin, Poland (isotypes).

### DNA extraction, PCR, cloning, and DNA sequencing

Genomic DNA for obtaining 45S and *rpb1* sequences was extracted from eight single spores of *E. infrequens* and each of the four potential new *Claroideoglomus* species. Details of the treatment of the spores prior to PCR, conditions and primers used for PCR, as well as cloning and sequencing of PCR products to obtain 45S sequences of the species are available in Krüger et al. (2009), Symanczik et al. (2014), and Błaszkowski et al. (2015b,c).

To obtain *rpb1* sequences of these five species, as well as *C. hanlinii, C. luteum*, and the species originally described as *G. walkeri*, a new set of primers was developed. The primer set consisted of two forward primers (RPB1-3F: GTC TTC GTG CAG TTT GGG A; RPB1-4F: CTA GGC CTG ATT GGA TGA T) and two reverse primers (RPB1-5R: ACG ATT TGT TTT GGT ACC AT; RPB1-5RN: TTC ATC TCA CCA TCA A). Characteristics of the primers and the PCR conditions are available in Błaszkowski et al. (2021a). To increase the chance to obtain a successful amplification of unknown sequences, the following approach was used to design the primers. All the possible 19-21 bases oligos obtained from the *rpb1* sequence available for *C. claroideum* were assessed according to the following criteria: (i) a perfect match with the *C*. *etunicatum* and *C. claroideum* sequences, (ii) at least one C/G in the 3′ end, and (iii) the highest number of matches among all the AMF *rpb1* sequences available (about 200) with maximum three mismatches tolerated if not in the last five residues at the 3′ end. Candidate oligos were evaluated with OligoAnalyzer for Tm and the presence of secondary structures.

Cloning and sequencing of *rpb1* PCR products were performed identically to those used to obtain 45S sequences (see above). Both 45S and *rpb1* sequences were deposited in GenBank (45S: MH590060, MH590061, MT722021–MT722024, ON950363–ON950390, and MT722034–MT722038; *rpb1*: MT733201–MT733210 and ON938320–ON938327).

### Phylogenomic analyses

Previous genome and transcriptome data phylogenies have shown two conflicting topologies with respect to Claroideoglomeraceae (Beaudet et al., 2018; Montoliu-Nerin et al., 2021). Therefore, we compiled three different datasets based on previously published data (Supplementary Table 1). The first dataset included data used by Beaudet et al. (2018) and Montoliu-Nerin et al. (2021), the second dataset included only Beaudet et al.’s (2018) data, and the third dataset included only genomes from the Montoliu-Nerin et al. (2021) analyses. Single-copy orthologs (SCOs) were identified with OrthoMCL (Li et al., 2003) using default settings, and only SCOs that were present in at least 50% of the taxa were included in the phylogenetic analyses. Individual SCOs were aligned with MAFFT 7.407 (Katoh and Standley, 2013). Poorly aligned regions were trimmed with trimAl 1.4.1 (Capella-Gutiérrez et al., 2009) with a gap threshold of 0.1. The individual alignments were concatenated using the script geneStitcher.py (Ballesteros and Hormiga, 2016). An ML phylogeny was inferred with IQ-TREE 2.0 (Nguyen et al., 2015) using ModelFinder (Kalyaanamoorthy et al., 2017) and searching for the best partition scheme. In addition, the individual SCO alignments were used to infer individual gene trees with IQ-TREE and then a phylogenetic inference with ASTRAL-III 5.7.3 (Zhang et al., 2018) was performed. The dataset that included only genome data used by Montoliu-Nerin et al. (2021) was then used to test the hypothesis that Claroideoglomeraceae is sister to Diversisporales and Glomeraceae. For this, we evaluated the support among individual gene trees for alternative branching orders and carried out a polytomy test in ASTRAL-III (Sayyari and Mirarab, 2018).

### Phylogenetic analyses

To clarify the status of *Albahypha* and *Entrophospora*, as well as to infer the position of the four potential new species, two sequence datasets were produced. The first consisted of sequences of the 45S segment, or part thereof, of our *E. infrequens* and the AMF, initially recognized as new species of *Claroideoglomus*, as well as all other sequenced species of these genera sensu Oehl et al. (2011b). *Claroideoglomus hanlinii*, a species described later (Błaszkowski et al., 2015a), was also included. The second dataset contained *rpb1* sequences available for the species of the first dataset and sequences from the genome of *C. candidum*. The sequences covered part of the fourth and fifth exons of the *rpb1* gene and the intron between them. In each of these datasets, the outgroup was represented by *Diversispora* species, and the datasets were aligned separately using MAFFT 7 with the E-INS-i strategy. Subsequently, the two alignments were used to manually produce a concatenated alignment, 45S+*rpb1*. The 45S sequences lacking the *rpb1* part were not removed from the alignment. An additional alignment was produced as follows: 45S+*rpb1*_G with three *Glomus* instead of *Diversispora* species in the outgroup to test the impact of this outgroup representing Glomeraceae, the sister family of Claroideoglomeraceae in the Glomerales sensu Schüßler and Walker (2010), on the topology and measures used in comparison of the trees generated in the analyses (described below).

The phylogenetic position of *Albahypha, E. infrequens*, and the four potential new species was reconstructed based on Bayesian inference (BI) and maximum likelihood (ML) phylogenetic analyses of the 45S, *rpb1*, 45S+*rpb1*, and 45S+*rpb1*_G alignments, performed via CIPRES Science Gateway 3.1 (Miller et al., 2010). The 45S alignment was divided into five partitions: 18S, ITS1, 5.8S, ITS2, and 28S. Five additional *rpb1* partitions were added in the alignments with the concatenated genes, as described in Błaszkowski et al. (2021b).

As suggested by Abadi et al. (2019), in both BI and ML analyses, GTR+I+G was chosen as the nucleotide substitution model for each nucleotide partition. Substitution models selected by ModelTest-NG 0.1.5 (Darriba et al., 2020) were also tested in ML analyses, but the trees obtained final loglikelihood values lower compared to those where GTR+I+G was used.

Four Markov chains were run over two million generations in MrBayes 3.2 (Ronquist et al., 2012), sampling every 2,000 generations, with a burn-in at 3,000 sampled trees. The ML phylogenetic tree inference was performed with RAxML-NG 1.0.1 (Kozlov et al., 2019) using a maximum likelihood/1,000 bootstrapping run and an ML estimated proportion of invariable sites and base frequencies. The alignments and tree files were deposited as Supplementary material.

We assumed that clades were supported when the Bayesian posterior probabilities and the ML bootstrap values were ≥0.95 and ≥70%, respectively. The generated phylogenetic trees were visualized and edited in TreeGraph 2 (Stöver and Müller, 2010). To evaluate possible conflicts between the genes, the topologies of the ML trees (collapsed at bootstrap values <70%) were compared. In addition, the trees were compared based on three measures, referred to the ingroup, which are as follows: (i) the number of species clades supported with BI ≥ 0.95 and ML ≥ 70%, (ii) mean BI and ML values for species clades when supported, (iii) mean supports of nodes with BI ≥ 0.95 and ML ≥ 70%, and (iv) the amount of resolution of each tree. The amount of resolution was calculated for the ingroup of the tree by dividing the number of significantly supported internal branches by the size of the ingroup (Thorley and Wilkinson, 2000).

To detect other possible findings of the potential new species, their 45S sequences were used as queries in BLASTn to retrieve more than 300 nucleotide sequences from GenBank. The sequences were selected according to the percentage of identity > 96.5, with at least one of the queries. The possible relatedness with any of the four species was assessed using an evolutionary placement algorithm-based (EPA) approach, performed with RAXML 8.2.10 (Berger et al., 2011). The sequences were aligned in MAFFT together with the 45S dataset, and the 45S+*rpb1* ML tree was used as reference support for the phylogenetic affiliation. GTRGAMMA model, the maximum likelihood estimation for the base frequencies, and the partition scheme previously described for the 45S alignment were used for the phylogenetic inference. The jplace output was used in Gappa (Czech et al., 2020) for the placement mass (likelihood weight ratio) accumulation of the placements of each sequence upward from the reference tree with the threshold = 0.9.

## Results

### General data and phylogeny

**FIGURE 1:**
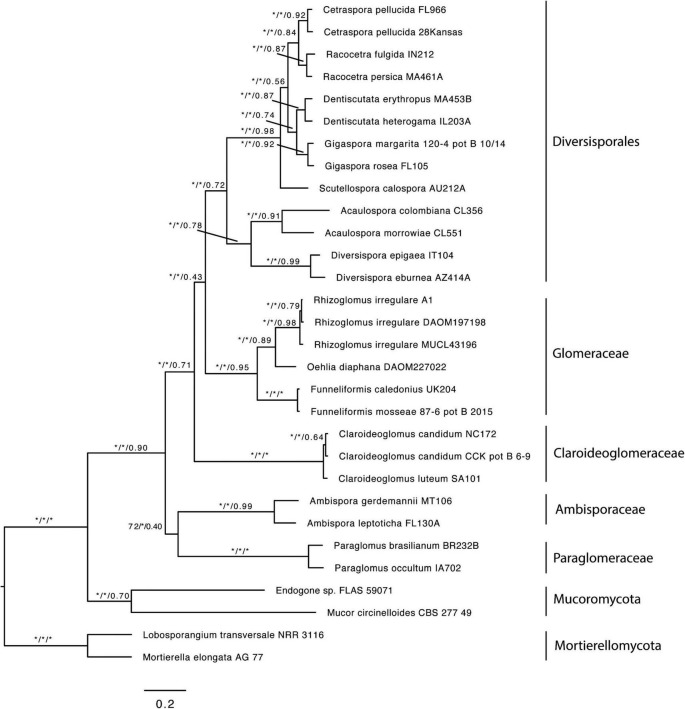
Best Maximum Likelihood phylogeny inferred with IQ-TREE from a concatenated alignment of 1260 single copy orthologs shared by at least 50% of the taxa. The same topology was recovered with ASTRAL III. Values near the branches correspond to bootstrap support (BS) from the ML analysis, local posterior probabilities (LPP), and quartet support from the ASTRAL analysis (BS/LPP/quartet support). Asterisks indicate maximum support (100 or 1.0). Mucoromycota and Mortierellomycota were used as outgroups. Bar indicates 0.2 expected change per site per branch.

**FIGURE 2:**
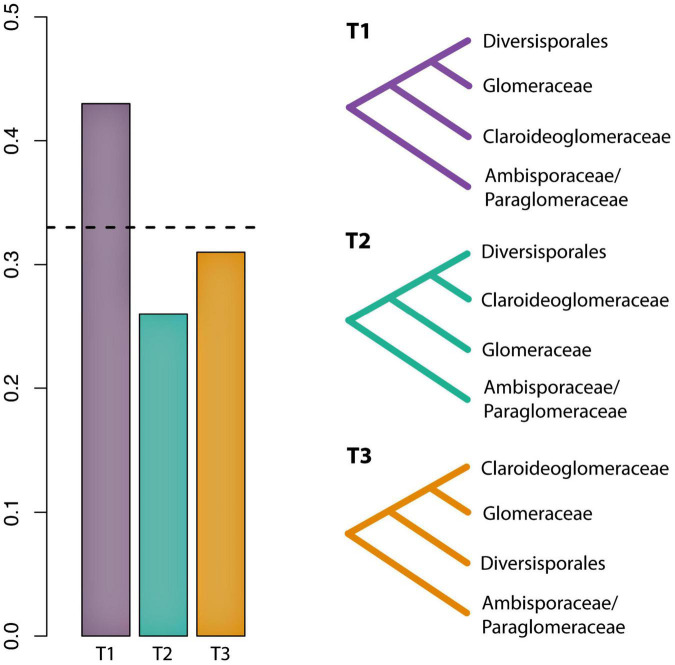
Evaluation of support among 1260 individual gene trees for alternative hypotheses of the phylogenetic placement of Claroideoglomeraceae. Bar graphs represent the gene tree quartet frequencies for three possible branching orders. The dashed horizontal line indicates the expectation of a hard polytomy.

The first dataset used for the phylogenomic analyses, which included data from Beaudet et al. (2018) and Montoliu-Nerin et al. (2021), contained 78 SCOs and recovered Claroideoglomeraceae as sister to Diversisporales and Glomeraceae (Supplementary Figure 1). Since Beaudet et al. (2018) showed Claroideoglomeraceae to be sister to Ambisporaceae and Paraglomeraceae based on analyses of spore transcriptomic data, we produced and analyzed a dataset that contained only these data. The analysis yielded 122 SCOs, and the phylogeny showed again Claroideoglomeraceae as sister to Diversisporales and Glomeraceae (Supplementary Figure 2). Finally, to obtain a higher number of SCOs and to perform a topology test, we excluded the Beaudet et al.’s (2018) dataset and analyzed only AM genome data used by Montoliu-Nerin et al. (2021). For this dataset, we obtained 1,260 SCOs present in at least 50% of the taxa, and the phylogenomic analysis also recovered Claroideoglomeraceae as sister to Diversisporales and Glomeraceae (Figure 1). Other phylogenomic analyses on every genome dataset consistently recovered Glomerales as polyphyletic, with Claroideoglomeraceae as sister to Diversisporales and Glomeraceae (data not shown). The quartet gene frequencies inferred with ASTRAL supported this relationship (q1 = 0.43, q2 = 0.26, q3 = 0.31) (Figure 2).

The phylogenetic position of *Albahypha, Claroideoglomus, E. infrequens*, and the four potential new species was reconstructed based on 115 sequences of the 45S segment or part thereof (28S) and 36 *rpb1* sequences. Of these, 57 were newly obtained (39 45S, 18 *rpb1*). The 45S and *rpb1* sequences characterized 14 and 10 species, respectively, of *Albahypha*/*Claroideoglomus*, including our four new species, as well as three *Diversispora* (*Glomus*) species that served as an outgroup. These sequences were used to prepare four alignments (45S, *rpb1*, 45S+*rpb1*, and 45S+*rpb1*_G) for BI and ML analyses. Data about the numbers of variable and parsimony informative sites of each alignment are presented in Table 1.

**TABLE 1:** Characteristics of the sequence alignments analyzed.

Name of alignment	No. of sequences	No. of fungal species	No. of base pairs	No. of variable sites	No. of parsimony informative sites
45S	106	14	1700	707	609
*rpb1*	27	10	948	291	270
45S+*rpb1*	106	14	2648	998	879
45S+*rpb1*_G	106	14	2665	975	864

**FIGURE 3:**
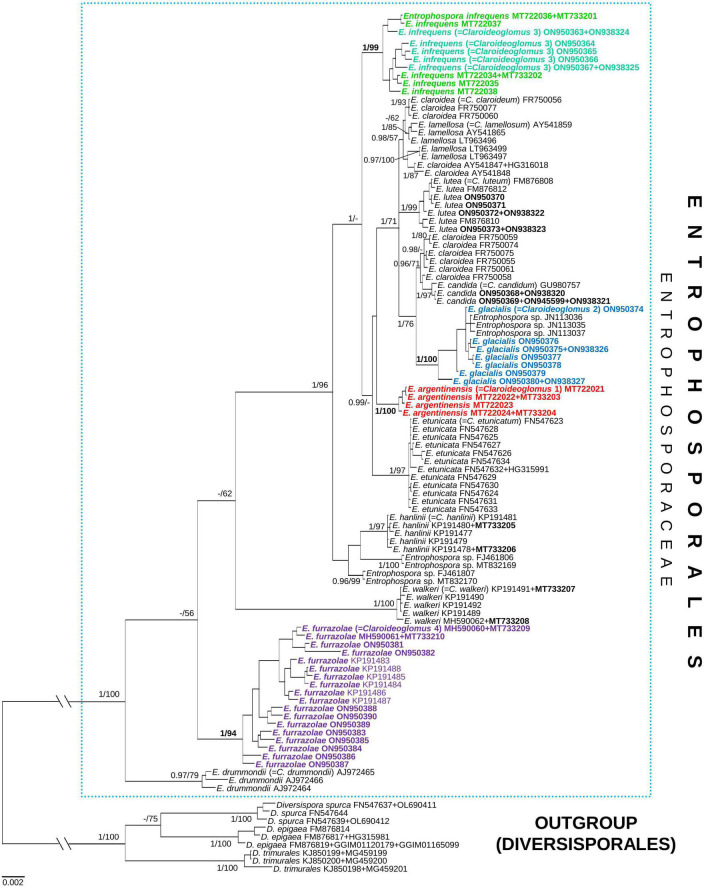
A 50% majority-rule consensus tree from the Bayesian analysis of 45S nuc rDNA sequences concatenated with *rpb1* sequences of *Claroideoglomus* 1, 2, 4 (newly described as *Entrophospora argentinensis, E. glacialis*, and *E. furrazolae*, respectively), *Claroideoglomus* 3 (a glomoid morph of the *E. infrequens* epitype), eight other species of *Claroideoglomus* sensu C. Walker and A. Schüßler, and three *Diversispora* species serving as outgroup. The former species names included in Entrophosporales are reported between brackets. The new species and the accession numbers of the sequences obtained in this study are in bold. The Bayesian posterior probabilities ≥0.90 and ML bootstrap values ≥50% are shown near the branches, respectively. Bar indicates 0.002 expected change per site per branch. The two basal branches were shortened to 20% in length to improve visibility (indicated by //).

Four phylogenetic trees summarizing the BI and ML analyses were reconstructed, named 45S, *rpb1*, 45S+*rpb1*, and 45S+*rpb1*_G (Figure 3 and Supplementary Figures 3–5, respectively). Small differences that occurred in the topologies of the trees were described below. In all trees, the number of supported species clades and the mean support values of species clades and nodes were identical or similar (Supplementary Table 2). Overall, the trees with concatenated genes performed slightly better than the 45S tree.

In all trees, the clades with *Claroideoglomus* 1–4 obtained full or almost full BI (= 0.99–1.0) supports (Figure 3 and Supplementary Figures 3–5). Also, the ML supports of these species in all trees were significant (≥70%), and most of them were very high or full.

In all trees, the glomoid *Claroideoglomus* 3 formed a mixed clade with the entrophosporoid *E. infrequens*, whose 45S and *rpb1* sequences were obtained from spores originating from our collection (Figure 3 and Supplementary Figures 3–5). In the 45S+*rpb1* and 45S trees, the glomoid *Claroideoglomus* 2 also grouped with *E. infrequens* (Figure 3 and Supplementary Figure 3), whose 28S sequences were obtained by Oehl et al. (2011b); no *rpb1* sequence of the fungus was available (pers. comm.). Hereafter, the two mixed clades will be referred to as *Claroideoglomus* 2 and *Claroideoglomus* 3.

In all trees, *Claroideoglomus* 1 and *Claroideoglomus* 3 occupied autonomous phylogenetic positions, except for the 45S tree, which showed an unsupported sister relationship of *Claroideoglomus* 1 with *C. etunicatum* (Figure 3 and Supplementary Figures 3–5). *Claroideoglomus* 2 formed a clade with *C. candidum* and six of the eleven analyzed sequences of *C. claroideum* in the 45S (not supported), 45S+*rpb1*, and 45S+*rpb1*_G trees. *Claroideoglomus* 4 was placed either in a strongly supported autonomous clade (in the 45S+*rpb1* and *rpb1* trees) or a BI-supported clade sister to *C. drummondii* (45S, 45S+*rpb1*_G). However, the ML supports of this sister relationship were insignificant (45S) or slight (45S+*rpb1*_G; ML = 76%).

Four other 28S sequences ascribed to *E. infrequens* formed a clade with or were accommodated in the neighborhood of *C. hanlinii* or clustered in an autonomous clade; none of the positions obtained support (Figure 3 and Supplementary Figures 3, 5). Although *Claroideoglomus* 4, *C. drummondii*, and *C. walkeri* formed a supported group corresponding to the genus *Albahypha* sensu Oehl et al. (2011b) in the 45S+*rpb1*_G tree, the clade comprising the other analyzed species was not supported (Supplementary Figure 5).

## Taxonomy

The results of the phylogenomic and phylogenetic analyses described above prompted us (i) to propose a new order, Entrophosporales, for Entrophosporaceae in place of Claroideoglomeraceae, (ii) to emend the descriptions of Glomerales, Entrophosporaceae, *Entrophospora*, and *E. infrequens*, (iii) to designate an epitype of *E. infrequens* with the morphology and phylogeny determined from analyses of the entrophosporoid *E. infrequens* morph and the glomoid morph preliminary named *Claroideoglomus* 3, which are grown in our collection, (iv) to introduce new nomenclatural combinations, and (v) to describe *Claroideoglomus* 1, 2, and 4 as *E. argentinensis* sp. nov., *E. glacialis* sp. nov., and *E. furrazolae* sp. nov., respectively.

### Description of a new order

**Entrophosporales** Błaszk., Sánchez-García, B.T. Goto, and Magurno, **ord. nov**.

MycoBank MB846043

*Type family*: Entrophosporaceae (Oehl and Sieverd.), emend. Błaszk., Sánchez-García, B.T. Goto, and Magurno.

*Diagnosis*: Producing entrophosporoid and glomoid spores or only one of these morphs. Entrophosporoid spores formed singly within the necks of sporiferous saccules, in soil, rarely in roots. Spores with two spore walls. Spore wall 1, forming the spore surface, composed of two short-lived to semi-permanent layers continuous with the neck and sporiferous saccule wall layers and a permanent, laminate, pigmented layer continuous with a wall of a cylindric to a funnel-shaped structure supporting the wall of the part of the neck directly connected with the sporiferous saccule and the wall of the saccule at its base; not extending into the part of the neck located distally to the sporiferous saccule, hence spores have only one persistent cicatrix, proximal to the sporiferous saccule, created when the opening connecting the interior of the spore with the lumen of the sporiferous saccule neck is closed by a plug made of the contents of the spore when fully formed; layer 1 stains dark in Melzer’s reagent. Spore wall 2, having no physical contact with spore wall 1, composed of three, hyaline, smooth layers: two thin, flexible layers enclosing a much thicker, semi-flexible layer; none of the layers stains in Melzer’s reagent. Glomoid spores formed at tips of sporogenous hyphae either branched from the sporiferous saccule wall and/or the sporiferous saccule neck of the entrophosporoid morph or continuous with extraradical mycorrhizal hyphae, occasionally intercalarily inside hyphae, rarely in roots, usually with one subtending hypha. Extraradical spores produced singly, rarely in clusters with few spores. Spores with one spore wall consisting of two to five layers; when multilayered, the innermost layer frequently is flexible to semi-flexible, <1.0 μm thick, colorless or brightly colored, and loosely associated with the inner surface of the penultimate, usually laminate, layer. One or two spore wall layers may stain in Melzer’s reagent. Subtending hypha with a hyaline, rarely brightly colored wall, always strikingly much lighter than the spore wall in colored spores, frequently conspicuously funnel- or bill-shaped at the spore base; subtending hyphal wall composed of layers continuous with spore wall layers. Pore closed by a septum connecting the inner surfaces of either the subtending hyphal wall or the spore wall, or septa connecting both these structures, at or slightly below the spore base, and frequently additionally by a septum continuous with the innermost flexible to semi-flexible spore wall layer. Entrophosporoid and glomoid species producing mycorrhiza with arbuscules, vesicles, as well as intra- and extraradical hyphae staining dark in Trypan blue.

### Emendation of Glomerales

Glomerales J.B. Morton and Benny, emend. Błaszk., B.T. Goto, and Magurno.

*Type family*: Glomeraceae Piroz. and Dalpé.

*Type genus*: *Glomus* Tul. and C. Tul. emend. Oehl, G.A. Silva, and Sieverd.

*Other genera*: *Dominikia* Błaszk., Chwat, and Kovács, *Microdominikia* Oehl, Corazon-Guivin, and G.A. Silva, *Kamienskia* Błaszk., Chwat, and Kovács, *Microkamieskia* Corazon-Guivin, G.A. Silva, and Oehl, *Orientoglomus* G.A. Silva, Oehl, and Corazon-Guivin, *Nanoglomus* Corazon-Guivin, G.A. Silva, and Oehl, *Septoglomus* Sieverd., G.A. Silva, and Oehl, *Rhizoglomus* Sieverd., G.A. Silva, and Oehl (= *Rhizophagus*), *Oehlia* Błaszk., Kozłowska, Niezgoda, B.T. Goto, and Dalpé, *Halonatospora* Błaszk., Niezgoda, B.T. Goto, and Kozłowska, *Sclerocarpum* B.T. Goto, Błaszk., Niezgoda, Kozłowska, and Jobim, *Epigeocarpum* Błaszk., B.T. Goto, Jobim, Niezgoda, and Magurno, *Funneliformis* C. Walker and A. Schüssler emend. Oehl, G.A. Silva, and Sieverd., *Funneliglomus* Corazon-Guivin, G.A. Silva, and Oehl, *Silvaspora* Błaszk., Niezgoda, B.T. Goto, Crossay, and Magurno, *Sclerocystis* Berk. and Broome.

*Diagnosis*: Spores formed at tips of sporogenous hyphae, occasionally intercalarily inside hyphae, and in roots, usually with one subtending hypha. Extraradical spores produced singly, in clusters with few to dozens of spores, compact unorganized glomerocarps (= sporocarps) with randomly distributed spores or organized glomerocarps with spores born terminally from hyphae developed radially from a central plexus of hyphae. Glomerocarps with or without a peridium. Spores with one spore wall consisting of one laminate layer or many layers; when multilayered, the innermost layer usually is laminate and thicker than the others. Subtending hypha with a wall concolorous with or slightly lighter than the spore wall, funnel-shaped, cylindrical, or constricted at the spore base; subtending hyphal wall composed of layers continuous with spore wall layers. Pore closed by thickening spore wall and subtending hyphal wall toward the center of the subtending hyphal lumen connecting it with the spore interior, a septum connecting the inner surfaces of the subtending hyphal wall slightly below the spore base, or the innermost spore wall layer at or slightly above the spore base. Mycorrhiza with arbuscules, vesicles, as well as intra- and extraradical hyphae staining dark in Trypan blue.

### Emendation of Entrophosporaceae, *Entrophospora, Entrophospora infrequens*, and new combinations

**Entrophosporaceae** (Oehl and Sieverd.), emend. Błaszk., Sánchez-García, B.T. Goto, and Magurno. = Entrophosporaceae Oehl and Sieverd., emend. Oehl, Sieverd., Palenz., and G.A. Silva. Mycotaxon 117:306, 2011. = Claroideoglomeraceae C. Walker and A. Schüßler. The *Glomeromycota* – a species list:21. 2010.

*Type genus*: *Entrophospora* R.N. Ames and R.W. Schneid., emend. Błaszk., Sánchez-García, Fernández, B.T. Goto, and Magurno.

*Diagnosis*: As that of Entrophosporales.

***Entrophospora*** R.N. Ames and R.W. Schneid., emend. Błaszk., Sánchez-García, Fernández, B.T. Goto, and Magurno. = *Albahypha* Oehl, G.A. Silva, B.T. Goto, and Sieverd., Mycotaxon 117:308. 2011. = *Claroideoglomus* C. Walker and A. Schüßler, emend. Oehl, Sieverd., B.T. Goto, and G.A. Silva, Mycotaxon 117:308. 2011. = *Claroideoglomus* C. Walker and A. Schüßler, The *Glomeromycota* – a species list:21. 2010.

*Type species*: *Entrophospora infrequens* (I.R. Hall) R.N. Ames and R.W. Schneid., emend. Błaszk., Sánchez-García, Fernández, B.T. Goto, and Magurno.

*Epitype*: Entrophosporoid morph, POLAND. Spores from a single-species culture established from spores extracted from a trap culture inoculated with a field-collected mixture of rhizosphere soil and root fragments of *J. communis* from a pine forest in inland sand dunes of Kampinos National Park (52*^o^*19′ N, 20*^o^*45′ E), July 1986, J. Błaszkowski (slide with spores no. 668–674, **isoepitypes** slides with spores no. 3870–3872, LPPDSE; 45S and *rpb1* sequences: MT722034–MT722038 and MT733201–MT733202, respectively, GenBank).

Glomoid morph, SPAIN. FORTUNA, MURCIA. Spores from a single-species culture established from spores extracted from a trap culture inoculated with a field-collected mixture of rhizosphere soil and root fragments of *L. sinuatum* from an initial community (38°06’57 N, 1°06’W), November 2009, F. Fernández (**epitype** slide with spores no. ZT Myc 0066907, **isoepitypes** slides with spores no. 3839–3846, LPPDSE); 45S and *rpb1* sequences: ON950364–ON950367 and ON938324–ON938325, respectively, GenBank.

*Diagnosis*: As that of Entrophosporales.

***Entrophospora infrequens*** R.N. Ames and R.W. Schneid., emend. Błaszk., Sánchez-García, Fernández, B.T. Goto, and Magurno.

*Diagnosis*: Differs from other *Entrophospora* species forming glomoid spores in spore morphometric features, phenotypic and histochemical properties of spore wall layers, as well as in nucleotide composition of sequences of the 45S nuc rDNA region and the *rpb1* gene.

**FIGURE 4:**
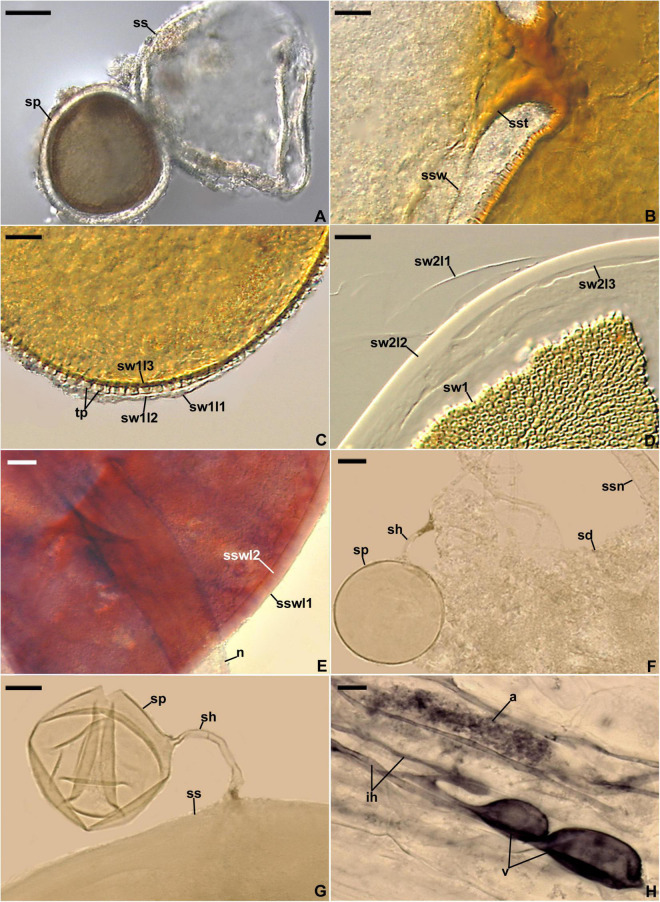
*Entrophospora infrequens*. **(A)** Entrophosporoid morph with spore (sp) formed inside the sporiferous saccule (ss). **(B)** Funnel-shaped structure, continuous with spore wall 1 layer 3, supporting the sporiferous saccule wall (ssw). **(C)** Spore wall 1 layers (sw1l) 1–3; swl1 is almost completely sloughed off; tooth-shaped projections (tp) in cross-view are visible. **(D)** Spore wall 1 (sw1) and spore wall 2 layers (sw2l) 1–3. **(E)** Sporiferous saccule wall layers (sswl) 1 and 2, and the neck (n) of sporiferous saccule. **(F)** Juvenile glomoid spore (sp) with subtending hypha (sh) developed from the sporiferous saccule neck (ssn); soil debris (sd) are indicated. **(G)** Juvenile glomoid spore (sp) with subtending hypha (sh) developed from sporiferous saccule (ss). **(H)** Mycorrhiza with arbuscule (a), vesicles (v), and intraradical hyphae (ih). **(A–D,F–H)** Spores and mycorrhizal structures in PVLG. **(E)** Sporiferous saccule in PVLG+Melzer’s reagent. **(A–H)** Differential interference microscopy. Scale bars: **(A)** = 50 μm, **(B–H)** = 10 μm.

*Description*: Producing entrophosporoid and glomoid glomerospores (= spores), or only one of these morphs. Entrophosporoid spores formed in soil, singly, arise blastically inside the neck of a sporogenous saccule (Figures 4A–H); golden yellow (5B8) to brownish orange (7C7); globose to subglobose; (95–)135(–175) μm diam, with two cicatrices. *Spores* with two spore walls having no physical contact with one another. Spore wall 1 composed of three layers. Layer 1, forming the spore surface, evanescent, hyaline, (1.5–)2.5(–4.5) μm thick, staining crayfish red (9B8) to cerise (12C8) in Melzer’s reagent, highly deteriorated or completely sloughed off in mature and older spores. Layer 2, semi-permanent, smooth, 2.8–6.0 μm thick, slowly degrading with age. Layer 3, permanent, laminate, golden yellow (5B8) to brownish orange (7C7); (1.5–)2.5(–4.5) μm thick, on the upper surface ornamented with tooth-shaped outgrowths, 1.8–3.8 × 1.0–3.0 μm, with a central depression in the upper surface. Layers 1 and 2 continuous with the neck and sporiferous saccule wall layers. Layer 3 continuous with a wall of a cylindric to funnel-shaped structure supporting the wall of the part of the neck directly connected with the sporiferous saccule and the wall of the saccule at its base; not extending into the part of the neck located distally to the sporiferous saccule. Spore wall 2 consisting of three permanent, hyaline layers. Layer 1, flexible, <0.5 μm thick, usually tightly adherent to layer 2, and, hence, frequently difficult to see. Layer 2, flexible to semi-flexible, coriaceous sensu Walker (1986), (5.5–)7.8(–10.0) μm thick. Layer 3 flexible, ∼0.5 μm thick, rarely separating from the lower surface of layer 2 in even vigorously crushed spores. None of the spore wall 2 layers 1–3 stains in Melzer’s reagent. *Cicatrices.* Visible as two scars in the region of contact between the spore and the saccule neck. A scar proximal to the saccule is a slightly depressed area, circular, 13.5–27.0 μm diam, to ellipsoidal, 13.5–23.0 × 16.0–27.0 μm, when seen in a plane view. A scar distal to the saccule is circular, 11.0–14.5 μm diam when observed in a plane view; it is usually completely sealed in mature spores and, therefore, invisible. *Sporiferous saccule* hyaline, consisting of a cylindric to slightly funnel-shaped neck, 21–33 wide, and a globose to egg-shaped, 100–170 × 130–190 μm, saccule, usually detached from mature spores. *Sporiferous saccule wall* composed of spore wall 1 layers 1–3. *Germination* unknown. *Mycorrhiza*. In single-species cultures with *P. lanceolata* and *Pueraria phaseoloides* (Roxb.) Benth. as the host plants, formed mycorrhiza with arbuscules, vesicles, and hyphae staining clearly in Trypan blue (Sieverding and Oehl, 2006; pers. observ.).

**FIGURE 5:**
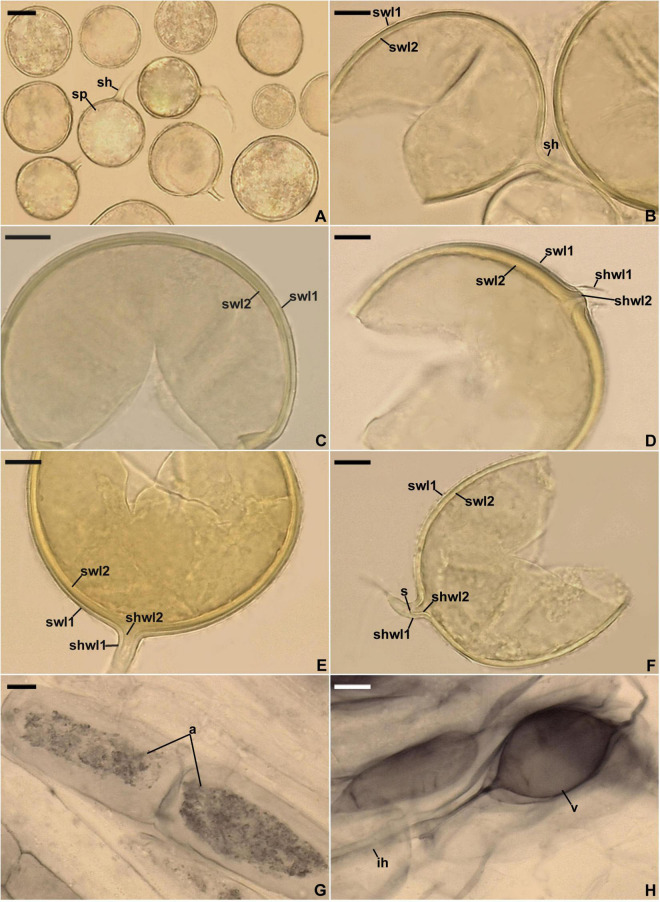
*Entrophospora infrequens*. **(A)** Intact glomoid spores (sp) with subtending hyphae (sh). **(B–F)** Spore wall layers (swl) 1 and 2 continuous with subtending hyphal wall layers (shwl) 1 and 2; a septum (s) continuous with swl2 in the lumen of the sh is indicated in **(F)**. **(G,H)** Arbuscules (a), intraradical hyphae (ih), and vesicles (v) in roots of *Plantago lanceolata* stained in 0.1% Trypan blue. **(A–D,G,H)** Spores and mycorrhizal structures in PVLG. **(E,F)** Spores in PVLG+Melzer’s reagent. **(A–H)** Differential interference microscopy. Scale bars: **(A)** = 20 μm, **(B–H)** = 10 μm.

Glomoid spores formed in soil, singly or in loose clusters with 2–3 spores, arise blastically at tips of sporogenous hyphae (Figures 5A,B,D–F) either branched from the sporiferous saccule wall and/or the sporiferous saccule neck of the entrophosporoid morph (Figures 4F,G), or continuous with extraradical mycorrhizal hyphae, occasionally intercalarily. *Spores* subhyaline to pastel yellow (3A4); globose to subglobose; (20–)50(–76) μm diam; rarely ovoid; 36–64 × 41–69 μm; with one subtending hypha (Figures 4F,G, 5A–F). *Spore wall* composed of two permanent layers (Figures 5B–F). Layer 1, forming the spore surface, uniform (not containing visible sublayers), semi-flexible, hyaline to yellowish white (3A2), (0.6–)1.1(–2.0) μm thick, tightly adherent to the upper surface of layer 2, rarely slightly deteriorated in its upper part (Figures 5B–F). Layer 2 laminate, semi-flexible, hyaline to pastel yellow (3A4), (1.2–)2.0(–3.4) μm thick; consisting of very thin, <0.5 μm thick, laminae, tightly adherent to each other (Figures 5B-F). In Melzer’s reagent, only layer 2 turns yellow (3A6; Figures 5E,F). *Subtending hypha* hyaline to pastel yellow (3A4); straight or recurved, usually slightly funnel-shaped, rarely cylindrical or slightly constricted at the spore base; (5.6–)7.1(–9.2) μm wide at the spore base (Figures 5A,B,D–F); not braking in crushed spores. *Wall of subtending hypha* subhyaline to pastel yellow (3A4); (1.4–)1.7(–2.8) μm thick at the spore base; consisting of two layers continuous with spore wall layers 1 and 2; subtending hyphal wall layer 2 formed up to 5.2 μm below the spore base (Figures 5D–F). *Pore* (2.0–)3.0(–3.8) μm wide at the spore base, usually open, rarely closed by a curved septum, 0.6–1.7 μm thick, continuous with spore wall layer 2 in mature spores (Figures 5B,D–F). Spore content of hyaline oily substance. *Germination* unknown. *Mycorrhiza*. In single-species cultures with *P. lanceolata* as the host plant, formed mycorrhiza with arbuscules, vesicles, as well as intra- and extraradical hyphae that stained dark [pale violet (16A3) to grayish violet (16E5)] in 0.1% trypan blue (Figures 4H, 5G,H).

*Ecology and distribution*: Entrophosporoid morph found associated with roots of many cultivated and uncultivated plant species growing in soils highly differing in pH and the content of nutrients, organic matter, and water (Sieverding and Oehl, 2006; Błaszkowski, 2012). Glomoid morph physically connected with an entrophosporoid morph grown in a single-species culture with the host plant *P. lanceolata* (Figures 4F,G) and probably lived in arbuscular mycorrhizal symbiosis with roots of *L. sinuatum* in the field, but no molecular analysis was performed to confirm this hypothesis. Reported as *C.* cf. *claroideum, E. infrequens*, Glomeromycota sp., and uncultured *Glomus* from Czech Republic, France, Peru, Poland, Spain, Switzerland, and USA (Supplementary Table 3).

### New combinations

***Entrophospora candida*** (Furrazola, Kaonongbua, and Bever) Błaszk., Niezgoda, B.T. Goto, and Magurno, **comb. nov**.

MycoBank MB836245

≡ *Glomus candidum* Furrazola, Kaonongbua, and Bever. Mycotaxon 113: 103. 2010 (basionym).

≡ *Claroideoglomus candidum* (Furrazola, Kaonongbua, and Bever) Oehl, G.A. Silva, and Sieverd. Mycotaxon 116:106. 2011.

***Entrophospora claroidea*** (N.C. Schenck and G.S. Sm.) Błaszk., Niezgoda, B.T. Goto, and Magurno, **comb. nov**.

MycoBank MB836246

≡ *Glomus claroideum* N.C. Schenck and G.S. Sm., Mycologia 74:84. 1982 (basionym).

≡ *Claroideoglomus claroideum* (N.C. Schenck and G.S. Sm.) C. Walker and A. Schüßler. The *Glomeromycota* – a species list:21. 2010.

***Entrophospora drummondii*** (Błaszk. and Renker) Błaszk., Niezgoda, B.T. Goto, and Magurno, **comb. nov**.

MycoBank MB836247

≡ *Glomus drummondii* Błaszk. and Renker. Mycological Research 110:559. 2006 (basionym).

≡ *Claroideoglomus drummondii* (Błaszk. and Renker) C. Walker and A. Schüßler. The *Glomeromycota* – a species list:22. 2010.

≡ *Albahypha drummondii* (Błaszk. and Renker) Sieverd., Oehl, B.T. Goto, and G.A. Silva. Mycotaxon 117:308. 2011.

***Entrophospora etunicata*** (W.N. Becker and Gerd.) Błaszk., Niezgoda, B.T. Goto, and Magurno, **comb. nov**.

MycoBank MB836248

*≡ Glomus etunicatum* W.N. Becker and Gerd. Mycotaxon 6:29. 1977 (basionym).

*≡ Claroideoglomus etunicatum* (W.N. Becker and Gerd.) C. Walker and A. Schüßler. The Glomeromycota – a species list:22. 2010.

***Entrophospora hanlinii*** (Błaszk., Chwat, and Góralska) Błaszk., Niezgoda, B.T. Goto, and Magurno, **comb. nov**.

MycoBank MB836249

*≡ Claroideoglomus hanlinii* Błaszk., Chwat, and Góralska. Mycological Progress 14:7. 2015 (basionym).

***Entrophospora lamellosa*** (Dalpé, Koske, and Tews) Błaszk., Niezgoda, B.T. Goto, and Magurno, **comb. nov**.

MycoBank MB836250

*≡ Glomus lamellosum* Dalpé, Koske, and Tews. Mycotaxon 43:289. 1992 (basionym).

*≡ Claroideoglomus lamellosum* (Dalpé, Koske, and Tews) C. Walker and A. Schüßler. The Glomeromycota – a species list:22. 2010.

***Entrophospora lutea*** (L.J. Kenn., J.C. Stutz, and J.B. Morton) Błaszk., Niezgoda, B.T. Goto, and Magurno, **comb. nov**.

MycoBank MB836251

*≡ Glomus luteum* L.J. Kenn., J.C. Stutz, and J.B. Morton. Mycologia 91:1090. 1999 (basionym).

*≡ Claroideoglomus luteum* (L.J. Kenn., J.C. Stutz, and J.B. Morton) C. Walker and A. Schüßler. The Glomeromycota – a species list:22. 2010.

***Entrophospora walkeri*** (Błaszk. and Renker) Błaszk., Niezgoda, B.T. Goto, and Magurno, **comb. nov**.

MycoBank MB836252

≡ *Glomus walkeri* Błaszk. and Renker. Mycological Research 110:563. 2006 (basionym).

≡ *Claroideoglomus walkeri* (Błaszk. and Renker) C. Walker and A. Schüßler. The *Glomeromycota* – a species list:22. 2010.

≡ *Albahypha walkeri* (Błaszk. and Renker) Sieverd., Oehl, B.T. Goto, and G.A. Silva. Mycotaxon 117:309. 2011.

### Description of new species

***Entrophospora argentinensis*** Błaszk., B.T. Goto, Magurno, Niezgoda, and Cabello, **sp. nov**.

Figures 6A–H, 7A,B.

**FIGURE 6:**
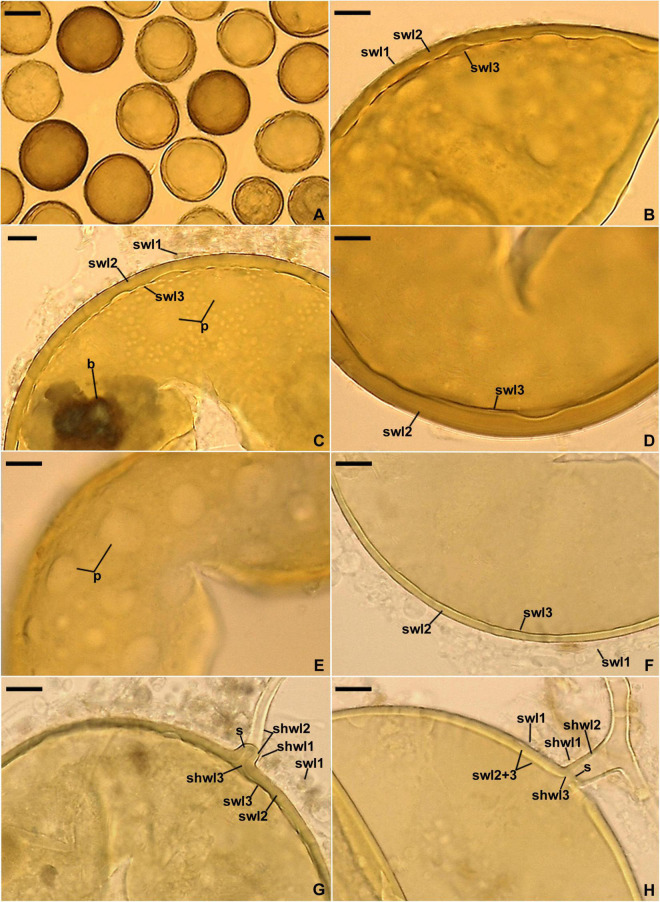
*Entrophospora argentinensis*. **(A)** Intact spores. **(B–D,F)** Spore wall layers (swl) 1–3; note the differences in thickness of the laminate swl2 in mature spores depicted in **(B–D)** and the equal thickness of swl2 in a young spore presented in **(F)**, as well as the birefringent (b) properties of layer 2 in polarized light visible in **(C)**. **(E)** Circular and ellipsoidal lighter patches (p) formed by the thinner areas of the laminate spore wall layer 2 seen in a plan view. **(G,H)** Subtending hyphal wall layers (shwl) 1–3 continuous with spore wall layers (swl) 1–3; note the highly deteriorated swl1 and a septum (s) in the subtending hyphal lumen formed by shwl2 and 3 continuous with swl2 and 3. **(A,D–H)** Spores in PVLG. **(B)** Spore in PVLG+Melzer’s reagent. **(A–H)** Differential interference microscopy. Scale bars: **(A)** = 50 μm, **(B–H)** = 10 μm.

**FIGURE 7:**
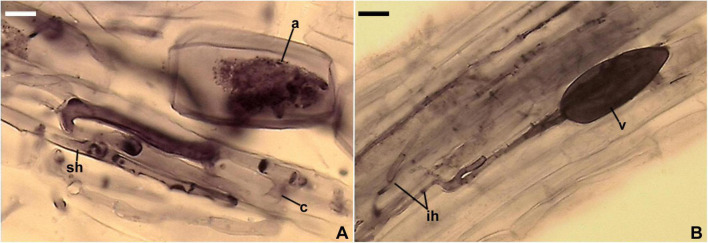
Mycorrhizal structures of *Entrophospora argentinensis* in roots of *Plantago lanceolata* stained in 0.1% Trypan blue. **(A)** Arbuscule (a), coiled (c) and straight (sh) intraradical hyphae. **(B)** Vesicle (v) and intraradical hyphae (ih). **(A,B)** In PVLG. **(A,B)** Differential interference microscopy. Scale bars: **(A)** = 10 μm, **(B)** = 20 μm.

MycoBank MB836244

*Typification*: ARGENTINA. TIERRA DEL FUEGO: Spores from a single-species culture established from spores extracted from a trap culture inoculated with a field-collected mixture of rhizosphere soil and root fragments of *D. flexuosa* and *Po. rigidifolia* from a steppe community (Mendoza et al., 2011), 1–15 Mar 2009, R. Mendoza (**holotype** slide with spores no. ZT Myc 61117, **isotypes** slides with spores no. 3694–3699, LPPDSE); 45S and *rpb1* sequences: MT722021–MT722024 and MT733203–MT733204, respectively, GenBank.

*Etymology*: *argentinensis* (Latin), referring to Argentina, where the species was originally found.

*Diagnosis*: Differs from other species of *Entrophospora* in having a laminate spore wall layer uneven in thickness in its different regions and nucleotide composition of sequences of the 45S nuc rDNA region and the *rpb1* gene.

*Description*: Glomerospores (= spores) glomoid, formed singly in soil (Figure 6A). Spores arising blastically at the tips of sporogenous hyphae (Figures 6G,H). *Spores* yellowish white (3A2) to dark yellow (4C8); globose to subglobose; (45–)95(–115) μm diam, rarely ovoid; 85–115 × 100–140 μm; with one subtending hypha (Figures 6A–H). *Spore wall* composed of three layers (Figures 6B–D,F–H). Layer 1, forming the spore surface, evanescent, semi-flexible, smooth in young spores, becoming roughened with age, occasionally completely sloughed off in older spores, hyaline to yellowish white (4A2), (0.8–)1.1(–1.5) μm thick when intact (Figures 6B–D,F–H). Layer 2 permanent, laminate, semi-flexible, yellowish white (3A2) to dark yellow (4C8), uneven in thickness when observed in a cross view, (1.0–)3.8(–7.5) μm thick in thinner regions, (2.4–)6.0(–10.0) μm thick in thicker regions; therefore, the lower edge of this layer is wavy, when observed in a plan view; thinner regions visible as circular, (1.2–)5.0(–13.0) μm diam or ellipsoidal, 5.2–7.0 × 7.4–14.8 μm, more or less evenly distributed lighter patches (Figures 6B–H); in young spores, layer 2 frequently uniform in thickness (Figures 6F,H); layer 2 with birefringent properties in polarized light, where smaller or larger areas, despite yellow-colored, turn almost black (Figures 6C,G). Layer 3 permanent, uniform (not divided into visible sublayers), semi-flexible, hyaline to light yellow (4A4), 0.8–1.3 μm thick, usually tightly adherent to the lower surface of layer 2, occasionally separating from layer 2 in vigorously crushed spores (Figures 6F,H). Layers 1–3 do not stain in Melzer’s reagent (Figure 6B). *Subtending hypha* yellowish white (3A2) to dark yellow (4C8) near the spore base, hyaline below the pigmented portion; straight or recurved, cylindrical or slightly funnel-shaped, rarely slightly constricted at the spore base; (6.2–)7.8(–8.8) μm wide at the spore base (Figures 6G,H); not braking in crushed spores. *Wall of subtending hypha* yellowish white (3A2) to dark yellow (4C8) up to 9.2 μm below the spore base, then hyaline; (2.0–)3.4(–4.8) μm thick at the spore base; consisting of three layers continuous with spore wall layers 1–3; subtending hyphal wall layer (shwl) 1 usually highly deteriorated or completely sloughed off even in young spores; shwl 3 present only at the spore base or extending up to 2.8 μm below the spore base (Figures 6G,H). *Pore* (0.8–)1.5(–3.4) μm wide at the spore base, occluded by a straight or curved septum continuous with a few innermost laminae of spore wall layer 2 and spore wall layer 3; septum 0.8–3.4 μm wide, 1.0–2.0 μm thick, positioned at or up to 4.2 μm below the spore base (Figures 6G,H). *Germination* unknown.

*Ecology and distribution*: In the field, associated with roots of *D. flexuosa* and *Po. rigidifolia* in Tierra del Fuego, Argentina. Forming mycorrhiza with arbuscules, vesicles, as well as intraradical and extraradical hyphae in single-species cultures with *P. lanceolata* as the host (Figures 7A,B); structures stained violet white (16A2) to deep violet (16E8) in 0.1% Trypan blue. Found as *Glomus* cf. *claroideum* associated with a spore of *E. infrequens* grown in trap culture inoculated with soil from an agricultural grassland of Switzerland and as trap-cultured glomeromycotan spore in the USA (Supplementary Table 3).

***Entrophospora glacialis*** Zubek, Niezgoda, B.T. Goto, Magurno, and Błaszk., **sp. nov.**

Figures 8A–H.

**FIGURE 8:**
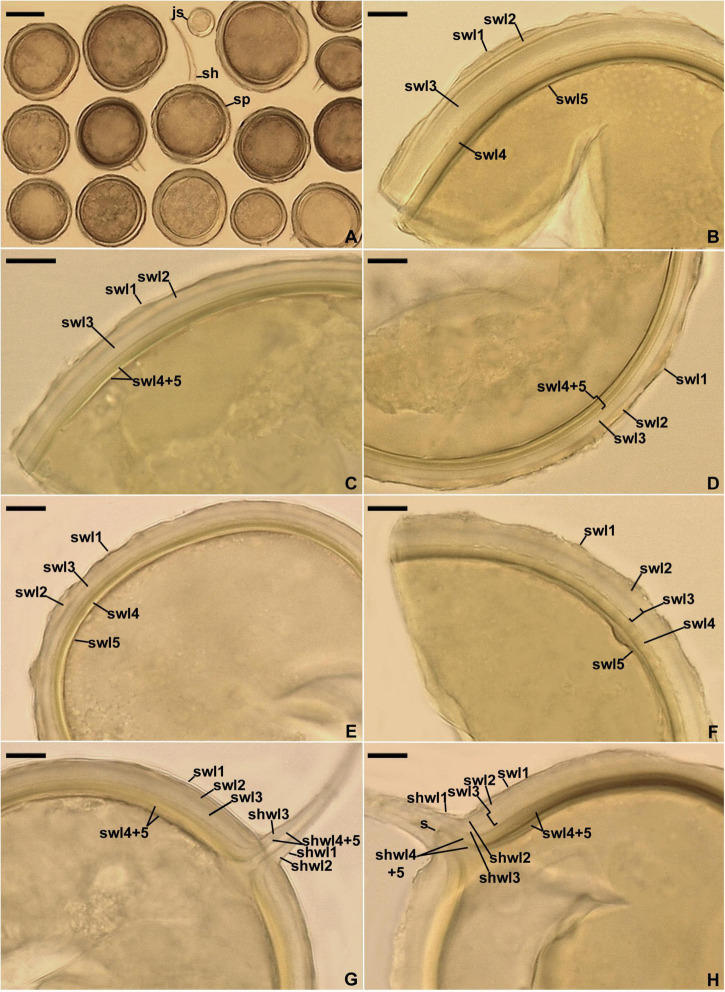
*Entrophospora glacialis*. **(A)** Intact spores (sp) with subtending hyphae (sh); juvenile spore (js) is indicated. **(B–F)** Spore wall layers (swl) 1–5. **(G,H)** Subtending hyphal wall layers (shwl) 1–5 continuous with spore wall layers (swl) 1–5; a septum (s) continuous with swl5 is indicated in **(H)**. **(A,E,G,H)** Spores in PVLG. **(B–D,F)** Spores in PVLG+Melzer’s reagent. **(A–H)** Differential interference microscopy. Scale bars: **(A)** = 50 μm, **(B–H)** = 10 μm.

MycoBank MB846045

*Typification*: SWEDEN. TARFALA VALLEY: Spores from a single-species culture established from spores extracted from a trap culture inoculated with a field-collected mixture of rhizosphere soil and root fragments of *F. vivipara, Po. alpina, S. herbacea, S. polaris*, and *Si. acaulis* from a glacier foreland of Isfallglaciarän (67°54′ N, 18°35′ E), 23 July 2019, P. Wietrzyk-Pełka and M. Węgrzyn (**holotype** slide with spores no. ZT Myc 0066908, **isotypes** slides with spores no. 3847–3859, LPPDSE); 45S and *rpb1* sequences: ON950374–ON950380 and ON938326–ON938327, respectively, GenBank.

*Etymology*: Latin, *glacialis*, referring to the glacial habitat, in which this new species was originally found.

*Diagnosis*: Differs from other *Entrophospora* species with glomoid spores in producing spores with a spore wall consisting of five permanent layers and nucleotide composition of sequences of the 45S nuc rDNA region and the *rpb1* gene.

*Description*: Glomerospores (= spores) glomoid, formed singly in soil, arise blastically at tips of sporogenous hyphae (Figures 8A–H) continuous with extraradical mycorrhizal hyphae. *Spores* yellowish white (4A2) to yellowish brown (5E8); globose to subglobose; (40–)91(–129) μm diam; rarely ovoid; 77–101 × 90–123 μm; with one subtending hypha (Figures 8A–H). *Spore wall* composed of five permanent layers (Figures 8B–H). Layer 1, forming the spore surface, uniform (not containing visible sublayers), semi-flexible, hyaline to yellowish white (4A2), (0.8–)1.3(–2.0) μm thick, tightly adherent to the upper surface of layer 2, occasionally with small, local thickenings rendering the spore surface slightly wavy when observed in a cross-section (Figures 8B–H). Layer 2 uniform, semi-flexible, yellowish white (4A2), (1.0–)1.8(–2.6) μm thick (Figures 8B–H). Layer 3 uniform, semi-flexible, hyaline to yellowish white (4A2), (2.0–)4.1(–9.0) μm thick (Figures 8B–H). Layer 4 laminate, semi-flexible, yellowish white (4A2) to yellowish brown (5E8), (2.4–)3.6(–5.0) μm thick, consisting of very thin, < 0.5 μm thick, laminae, tightly adherent to each other (Figures 8B–H). Layer 5 uniform, flexible to semi-flexible, (1.0–)1.1(–1.2) μm thick, usually slightly separating from the lower surface of the laminate layer 4 (Figures 8B–H). None of the spore wall layers stains in Melzer’s reagent. *Subtending hypha* hyaline to yellowish white (4A2); straight or recurved, usually slightly funnel-shaped, rarely cylindrical or slightly constricted at the spore base; (8.4–)10.8(–14.2) μm wide at the spore base (Figures 8A–H); not braking in crushed spores. *Wall of subtending hypha* hyaline to yellowish white (4A2); (3.8–)4.8(–6.4) μm thick at the spore base; consisting of five layers continuous with spore wall layers 1–5; subtending hyphal wall layers 4 and 5 formed up to 6.1 μm below the spore base (Figures 8A–H). *Pore* (1.0–)1.4(–2.0) μm wide at the spore base, usually open, rarely closed by a curved septum, 0.7–1.1 μm thick, continuous with spore wall layers 4 and 5 (Figures 8G,H). Spore content of hyaline oily substance. *Germination* unknown.

*Ecology and distribution*: In the field, present under an initial community consisting of cryptogamic species, as well as *F. vivipara, Po. alpina, S. herbacea, S. polaris*, and *Si. acaulis* in a glacier foreland of Sweden. No molecular analysis was performed to reveal which of these species harbored *E. glacialis*. In single-species cultures with *P. lanceolata* as the host plant, *E. glacialis* formed mycorrhiza with arbuscules, vesicles, as well as intra- and extraradical hyphae that stained dark [pale violet (16A2) to deep violet (16D8)] in 0.1% trypan blue. Found as *C. claroideum, C.* cf. *claroideum, E. infrequens, G. luteum*, Glomeromycota sp., and uncultured *Glomus* in cultivated and uncultivated sites, including submerged and contaminated habitats, in Brazil, China, Japan, Netherlands, Norway, Poland, Switzerland, and USA (Supplementary Table 3).

***Entrophospora furrazolae*** Magurno, Niezgoda, B.T. Goto, and Błaszk., **sp. nov.**

Figures 9A–H.

**FIGURE 9:**
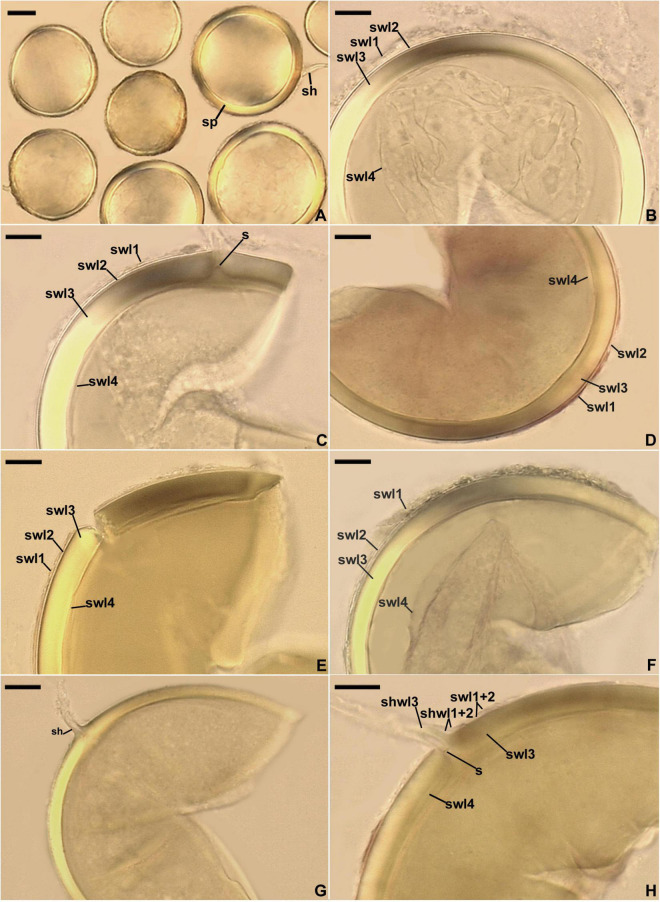
*Entrophospora furrazolae*. **(A)** Intact spores (sp) with subtending hyphae (sh). **(B–F)** Spore wall layers (swl) 1–4; a septum (s) continuous with swl4 is indicated in **(C)**. **(G,H)** Subtending hypha (sh) with subtending hyphal wall layers (shwl) 1–3; a septum (s) continuous with swl4 is indicated in **(H)**. **(A–C)** Spores in PVLG. **(D–H)** Spores in PVLG+Melzer’s reagent. **(A–H)** Differential interference microscopy. Scale bars: **(A)** = 20 μm, **(B–H)** = 10 μm.

MycoBank MB 846048

*Typification*: POLAND. LUBLINIEC, SILESIAN UPLAND: Spores from a single-species culture established from spores extracted from a trap culture inoculated with a field-collected mixture of rhizosphere soil and root fragments of *Po. trivialis* from a plant community in the unpolluted shore of Kokotek pond II (50°37′ N, 18°43′ E), July 2016, Monika Malicka (**holotype** slide with spores no. ZT Myc 0066909, **isotypes** slides with spores no. 3860–3869, LPPDSE); 45S and *rpb1* sequences: MH590060, MH590061, ON950381–ON950390, and MT733209–MT733210, respectively, GenBank.

*Etymology*: Latin, *furrazolae*, in honor of Eduardo Furrazola, in recognition of his important contribution to studies of arbuscular mycorrhizal fungi. This recognition comes after the premature death caused by COVID-19 in 2021.

*Diagnosis*: Differs from other *Entrophospora* species with glomoid spores in formation of spores with a spore wall containing three permanent layers and two layers staining in Melzer’s reagent, as well as in nucleotide composition of sequences of the 45S nuc rDNA region and the *rpb1* gene.

*Description*: Glomerospores (=spores) glomoid, formed singly in soil, arise blastically at tips of sporogenous hyphae (Figures 9A,C,G,H) continuous with extraradical mycorrhizal hyphae. *Spores* hyaline to yellowish white (4A2); globose to subglobose; (60–)72(–87) μm diam; rarely ovoid; 50–57 × 60–74 μm; with one subtending hypha (Figures 9A–H). *Spore wall* composed of four layers (Figures 9B–F,H). Layer 1, forming the spore surface, mucilaginous, short-lived, flexible, hyaline, (0.6–)0.8(–1.0) μm thick, usually highly deteriorated or completely sloughed off in mature spores (Figures 9B–F,H). Layer 2 uniform (without visible sublayers), permanent, flexible to semi-flexible, hyaline, (0.8–)1.0(–1.6) μm thick, tightly adherent to the upper surface of layer 3, not separating from this layer in even vigorously crushed spores (Figures 9B–F,H). Layer 3 laminate, permanent, semi-flexible, hyaline to yellowish white (4A2), (2.4–)6.4(–10.2) μm thick, consisting of very thin, <0.5 μm thick, laminae, tightly adherent to each other (Figures 9B–F,H); layer 3 has birefringent properties in polarized light, in which smaller or larger colorless or yellowish white (4A2) fragments of this layer turn almost black (Figures 9B,C,E,F). Layer 4 flexible, permanent, hyaline, (0.6–)0.8(–1.0) μm thick, usually separating from the lower surface of the laminate layer 3 in even moderately crushed spores (Figures 9B–F,H). In Melzer’s reagent, spore wall layers 1 and 4 frequently stain reddish white (10A2) to grayish rose (11B4) and reddish white (8A2) to pastel pink (11A3), respectively, but these staining reactions do not appear in all spores (Figures 9D–H). *Subtending hypha* hyaline; straight or recurved, usually slightly funnel-shaped, rarely cylindrical or slightly constricted at the spore base; (3.6–)5.3(–7.4) μm wide at the spore base (Figures 9A,C,G,H); frequently braking at the base of crushed spores. *Wall of subtending hypha* hyaline; (1.6–)2.3(–3.5) μm thick at the spore base; consisting of three layers continuous with spore wall layers 1–3; subtending hyphal wall layer (shwl) 1 usually highly deteriorated or completely sloughed off in mature spores, thereby difficult to see or invisible; shwl2 usually present only at the spore base, rarely starting developing up to ca. 6 μm below the spore base; shwl3 gradually thickening beginning from ca. 9 μm below the spore base (Figure 9H). *Pore* (0.8–)1.0(–1.4) μm wide at the spore base, closed by a curved septum, 0.6–1.0 μm thick, continuous with spore wall layer 4; the septum usually located at the center of the laminate spore wall layer 3 when observed in a cross section (Figures 9C–H). Spore content of hyaline oily substance. *Germination* unknown.

*Ecology and distribution*: In Oman, *E. furrazolae* probably lived in the field in arbuscular mycorrhizal symbiosis with *Pr. cineraria* in a site characterized by hyper aridity, but no molecular analysis was performed to confirm this hypothesis. In Poland, *E. furrazolae* was associated with *Po. trivialis* in an unpolluted natural site, as indicated by NGS sequencing of partial 28S PCR amplicons obtained from DNA extracted from rhizosphere soil and root samples of this plant species (Malicka et al., 2022). In single-species cultures with *P. lanceolata* as the host plant, *E. furrazolae* formed mycorrhiza with arbuscules and intra- and extraradical hyphae that stained bluish-white (20A2) to light blue (20A5) in 0.1% trypan blue. Found as *C. drummondii*, as well as uncultured Claroideoglomeraceae, *Glomus*, and Glomeromycota in undisturbed and degraded soils of Czech Republic, China, Spain, and Switzerland (Supplementary Table 3).

## Discussion

Overall, the results of phylogenomic, phylogenetic, and morphological analyses presented in this study led to the following statements. First, the glomoid spore-producing species of the genus *Claroideoglomus* with the type species *C. claroideum* in the monogeneric family Claroideoglomeraceae sensu Schüßler and Walker (2010) represent a new order in the Glomeromycota, here described as Entrophosporales. Second, the entrophosporoid morph morphologically conspecific with *E. infrequens* represents a group of cryptic and potential dimorphic species widely distributed in the world. Third, our hypothesis was confirmed: the glomoid spore-producing AMF, preliminary named as *Claroideoglomus* 1, 2, and 4, originating from Argentina, Poland, Oman, and Sweden, are actually new species, described here as *E. argentinensis*, *E. glacialis*, and *E. furrazolae*. Finally, Redecker et al.’s (2013) conclusion that *Albahypha* is an unsupported taxon was confirmed (Figure 3 and Supplementary Figures 3–5).

Previous phylogenomic analyses have suggested that the order Glomerales is polyphyletic (Beaudet et al., 2018; Montoliu-Nerin et al., 2021), but the phylogenetic position of Claroideoglomeraceae was not clearly established. When data from Beaudet et al. (2018) and Montoliu-Nerin et al. (2021) were combined, we recovered the same topology as in Montoliu-Nerin et al. (2021). The topology, showing Claroideoglomeraceae sister to Diversisporales and Glomeraceae, was consistently recovered in all other analyses, even when only the Beaudet et al.’s (2018) data were used (Figure 1 and Supplementary Figures 1, 2). However, when combining all the data, we were able to extract only 78 SCOs present in at least 50% of the taxa (vs. 1260 SCOs), which could be due to the low genome completeness of the transcriptomic data presented in Beaudet et al. (2018).

In all phylogenomic analyses, we included only a few members of Mortierellomycota and Mucoromycota as outgroups. This was because the phylogenetic relationship among the three phyla was not the focus of the present work, but rather the phylogenetic placement of the former Claroideoglomeraceae with respect to the rest of Glomeromycota. This (i) resulted in better taxonomic resolution at the phylum level (Glomeromycota), (ii) provided consistent support for the phylogenetic placement of the former Claroideoglomeraceae as sister to a clade formed by Diversisporales and Glomeraceae, and, consequently, (iii) supported the need to transfer the Claroideoglomeraceae from Glomerales to a new order, Entrophosporales. The name of the order followed the synonymization of Claroideoglomeraceae with Entrophosporaceae because *E. infrequens* was described before *C. claroideum* (Ames and Schneider, 1979; Schenck and Smith, 1982). Through this, the order Glomerales remained with one family, Glomeraceae. Furthermore, the comparisons of the phylogenetic trees reconstructed from analyses with concatenated genes using *Glomus* (Glomerales after emendation) or *Diversispora* (Diversisporales) as alternative outgroups, despite some differences in the topology (Figure 3 and Supplementary Figures 3, 5), did not lead to different conclusions about the status of the new species, and *Albahypha* was not a valid generic taxon.

Both 45S, 45S+*rpb1*, and 45S+*rpb1*_G trees showed a group of taxa, formerly *Claroideoglomus* sensu Oehl et al. (2011b), characterized by short branches and a few deep-supported nodes (Figure 3 and Supplementary Figures 3, 5), a pattern that resembles an evolutionary radiation event, and a group of three sister taxa, referable to the former *Albahypha* sensu Oehl et al. (2011b), characterized by longer branches and different relationships depending on the dataset used. In the former *Claroideoglomus* clade, the species *E. claroidea* and *E. lamellosa* were always unresolved, as also shown in Delavaux et al. (2022) for the former species, a situation coherent with the evolutionary radiation hypothesis. The *Albahypha* clade occurred only in the 45S+*rpb1*_G phylogeny, where the former *Claroideoglomus* clade was affected by insufficient BI and ML supports. The presence of a supported *Albahypha* clade in the 45S+*rpb1*_G phylogeny probably resulted from long-branch attraction (LBA) rather than true close relationships.

Our research significantly enriched the knowledge of Glomeromycota by showing the dimorphism and cryptic nature of the species called *E. infrequens*, previously known only for its entrophosporoid morph with a distinctive and uniform morphology. First, both nuc rDNA and *rpb1* sequences of one of the analyzed morphs, here designated as an epitype of *E. infrequens* from spores found in Poland, clustered with sequences of a Spanish glomoid morph preliminary named *Claroideoglomus* 3 (Figure 3 and Supplementary Figures 3–5). The sequences from the two isolates were obtained in different years and from two different laboratories, so we ruled out the possibility that our findings could be biased by PCR contamination. Second, sequences of a Swedish glomoid morph (*Claroideoglomus* 2/*E. glacialis*) formed a mixed clade with sequences obtained from *E. infrequens* identified in Switzerland (Oehl et al., 2011b). Third, the FJ461806, FJ461807, MT832169, and MT832170 sequences of *E. infrequens* originating from the U.S. Indiana and California states were placed in unresolved positions of the trees. Finally, the entrophosporoid morph of the *E. infrequens* epitype was shown to be physically connected with glomoid spores (Figures 4F,G). Consequently, the discovery of the dimorphic and cryptic nature explained the early inexplicable localization of sequences obtained from this entrophosporoid morph in different phylogenetic tree clades and unraveled the inconsistent opinion on the taxonomic status of *E. infrequens* sensu Ames and Schneider (1979).

The dimorphic behavior of AMF has been observed almost exclusively in acaulosporoid spore-producing species of the genera *Ambispora* and *Archaeospora*, whose second morph were glomoid spores (Goto et al., 2008; Palenzuela et al., 2011; Bills and Morton, 2015; Oehl et al., 2019). The exceptions were *Ar. ecuadoriana*, which formed acaulosporoid, entrophosporoid, and glomoid spores (Schüßler and Walker, 2019), as well as *Am. callosa* sensu Walker et al. (2007), synonymized with *Am. leptoticha* (Bills and Morton, 2015), whose phylogeny was reconstructed from analyses of DNA originating from the only known glomoid morph of this species, originally described as *G. callosum* (Sieverding, 1988).

According to Lücking et al. (2021), cryptic speciation may be caused by the delay of phenotypic divergence relative to phylogenetic divergence because, e.g., (i) species may have diverged too recently to have also diverged in phenotype and (ii) the forces at work were selective and prevented phenotypes from diverging. This may explain the phenotypic indistinctness of the entrophosporoid morph representing different glomoid spore-producing *Entrophospora* species (Figures 3, 5–8 and Supplementary Figures 3–5). On the other hand, the massive construction of entrophosporoid spores, consisting of two thick walls with four permanent components, may have acted as a physical barrier that prevented the production of other entrophosporoid phenotypes due to the influence of the external environment.

Dimorphism is not the persistent behavior of dimorphic AMF species which, for undefined reasons, produced either both morphs (e.g., acaulosporoid and glomoid by *Am. leptoticha*) or only one of them (glomoid by another strain of *Am. leptoticha*) even over many propagation cycles (Bills and Morton, 2015). More (1998) concluded that the dimorphism in even extensively studied fungal groups was influenced or governed by a wide range of unspecific metabolic and environmental factors.

Thus, the cryptic nature and the dimorphic abilities of the entrophosporoid morph implied treating the glomoid spore-producing species of the genus *Claroideoglomus* sensu Schüßler and Walker (2010) as members of the newly emended genus *Entrophospora*, in accordance with the new combinations introduced in the section “Taxonomy”.

Like the phylogenetic analyses, morphological comparisons also showed that the relationships between the three new glomoid spore-producing *Entrophospora* species and the glomoid morph of the epitype of *E. infrequens* versus all previously sequenced *Entrophospora* species are bland or very weak. None of the known *Entrophospora* species produces spores (i) with a laminate spore wall layer, which is uneven in thickness in its different regions, as in *E. argentinensis* (Figures 6B–E,G), (ii) so strongly resembling spores of species of *Dominikia* and related genera in their frequent formation in loose clusters, appearance, size, pigmentation, spore wall structure, as well as in the phenotypic and histochemical properties of components of this wall (Błaszkowski et al., 2021b), as the glomoid spores of *E. infrequens* (Figures 4F,G, 5A–F), (iii) with a spore wall consisting of five permanent layers, as in *E. glacialis* (Figures 8B–H), and (iv) with a spore wall containing three permanent layers, of which two stain in Melzer’s reagent, except for *E. furrazolae* (Figures 9B–F,H).

The poorly and ambiguously supported relationship of *E. glacialis* to *E. claroidea* and *E. candida* (Figure 3 and Supplementary Figures 3, 5) somewhat confirms their morphological similarity. Spores of *E. glacialis* and *E. claroidea* (i) are surrounded by a halo produced by colorless or slightly colored spore wall layers surrounding the main structural, much darker colored, laminate spore wall layer and (ii) have a spore wall with an innermost layer that is flexible to semi-flexible, colorless, thin, and usually separates from the lower surface of the laminate layer in crushed spores (Figures 8A–H; Błaszkowski et al., 2021b). However, in *E. claroidea*, the halo is produced by two impermanent layers, frequently highly or completely sloughed off in mature specimens, of which the outermost layer 1, forming the spore surface, stains in Melzer’s reagent. The innermost *E. claroidea* spore wall component is layer 4. Instead, in *E. glacialis* the halo is formed by three permanent layers (layers 1–3), remaining intact even in old spores, and none of the five-layered spore wall layers reacts in Melzer’s (Figures 8A–H). In addition, *E. claroidea* spores are clearly lighter in color, not reaching the shade of brown of *E. glacialis* spores at maturity.

Spores of *E. candida* also have a halo when all spore wall layers are present (Furrazola et al., 2010). However, according to the original description, spores of this species compared to those of *E. glacialis* are much lighter (white, rarely pale yellow) and 1.2- to 2.2-fold larger when globose, have only a two-layered and ∼ 1.4-fold thinner spore wall, as well as may have an up to 1.5-fold wider subtending hypha at the spore base.

Although unsupported, the appearance of a sister relationship of *E. argentinensis* to *E. etunicata* in the 45S tree (Supplementary Figure 3) and the neighborhood of these species in the 45S+*rpb1, rpb1*, and 45S+*rpb1*_G trees (Figure 3 and Supplementary Figures 4, 5) call for a comparison of their morphology. As mentioned above, none of the glomoid spore-producing *Entrophospora* species, including *E. etunicata*, has a laminate spore wall layer that is uneven in thickness (Błaszkowski, 2012; Błaszkowski et al., 2015a). In addition, the spore wall of *E. etunicata* consists of two layers only, lacking the semi-flexible spore wall layer 3 of *E. argentinensis* (Figures 6B–D,F–H), and the spore wall layer 1 of *E. etunicata* stains in Melzer’s reagent (Becker and Gerdemann, 1977; Stürmer and Morton, 1997; Błaszkowski, 2012). In *E. argentinensis*, none of the spore wall layers reacts in this reagent (Figure 6B). Moreover, the pore of the spore subtending hypha of *E. etunicata* is closed by a septum continuous with the innermost laminae (sublayers) of the laminate spore wall layer 2, and that of *E. argentinensis* is closed by the innermost laminae of the laminate spore wall layer 2 and a septum continuous with the innermost spore wall layer 3 (Figures 6G,H). Finally, globose *E. etunicata* spores may be 1.4-fold larger.

Morphologically, *E. furrazolae* may be confused with *E. drummondii*, a sister species indicated in the 45S and 45S+*rpb1*_G trees (Figure 3 and Supplementary Figure 3). In both species, the flexible, thin innermost spore wall layer, forming a septum in the subtending hypha, stains in Melzer’s reagent (Figures 9D–H; Błaszkowski et al., 2006; Błaszkowski, 2012), a property unknown in other glomoid spore-producing species. However, in *E. drummondii*, this staining reaction occurs only in spore wall layer 3, and in *E. furrazolae* it appears in spore wall layer 4, absent in the spore wall of the former species, and spore wall layer 1 (Figures 9D–H). In addition, the spore wall of *E. drummondii* is 1.4- to 1.9-fold thinner, and the mature spores of this species are always slightly darker colored.

We omitted in our analyses the genus *Viscospora* included in the Entrophosporaceae defined by Oehl et al. (2011b) because (*i*) its sole species, *V. viscosa*, originally described as *G. viscosum* (Walker et al., 1995), is provided with only one 18S nuc rDNA sequence and (*ii*) Redecker et al. (2013) found that *V. viscosa* belongs to the genus *Septoglomus*. However, morphologically *V. viscosa* does not match any *Septoglomus* species. Therefore, more robust molecular proofs are needed to clarify the phylogenetic position of *V. viscosa*. In addition, our analyses included the MT733209 and MT733210 *rpb1* sequences erroneously ascribed to *C.* cf. *drummondii* by Błaszkowski et al. (2021a) instead of to the new *E. furrazolae*.

We recommend for the upcoming works involving species in the Entrophosporales that (i) *E. infrequens*-like culture collections should be checked carefully to detect the possible presence of dimorphism, (ii) the morphology of *E. infrequens*-like and/or glomoid-like spores should be carefully described to highlight any possible difference not yet detected, and (iii) descriptions of new species based on *E. infrequens*-like cultures should be based on strong molecular data.

## Data availability statement

The datasets presented in this study can be found in online repositories. The names of the repository/repositories and accession number(s) can be found below: NCBI MH590060, MH590061, MT722021–MT722024, ON950363–ON950390, MT722034–MT722038, MT733201–MT733210, and ON938320–ON938327.

## Author contributions

JB, MS-G, BG, LC, FM, MM, PM, PN, FF, MC, AV, MNA, WB, and SZ: material preparation, data collection, and analysis. JB, MS-G, BG, LC, and FM: conceptualization. JB, MS-G, BG, FM, and PN: methodology. JB, MS-G, BG, FM, MM, PM, PN, SS, RM, and SZ: formal analysis, investigation, and writing— review and editing. JB, BG, and FM: writing—original draft preparation. BG, FM, PN, and SZ: funding acquisition. JB, FM, and PN: resources. JB: writing first draft of the manuscript and supervision. All authors commented on previous versions of the manuscript, contributed to the study conception and design, read, and approved the final manuscript.
