# Supplementary figures and images for: A new order, Entrophosporales, and three new Entrophospora species in Glomeromycota
Source: Front Microbiol. 2022 Nov 29;13:962856. doi: 10.3389/fmicb.2022.962856 (PMC9835108; doi:10.3389/fmicb.2022.962856)

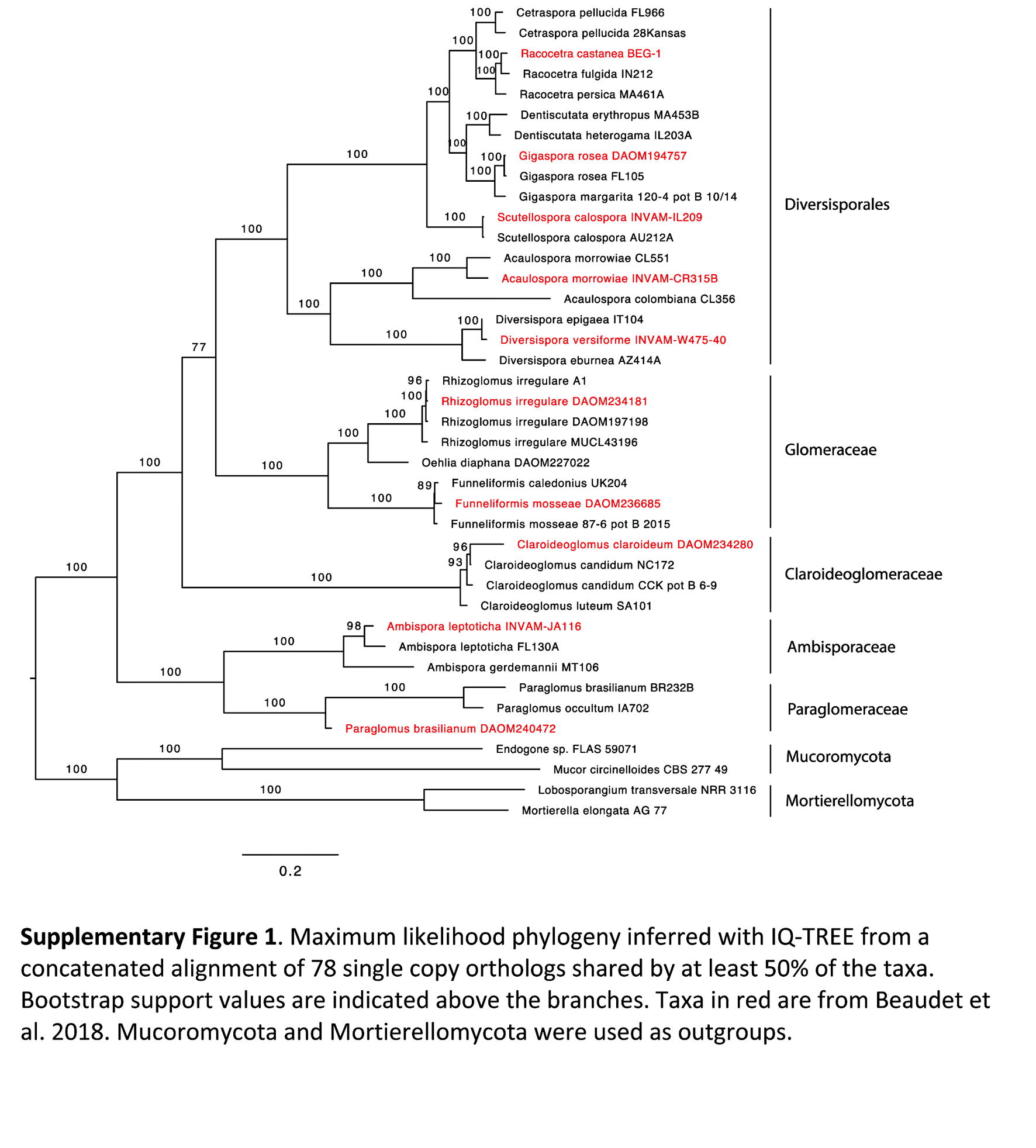

Supplement: Supplementary file 1 [file Image_1.TIF]

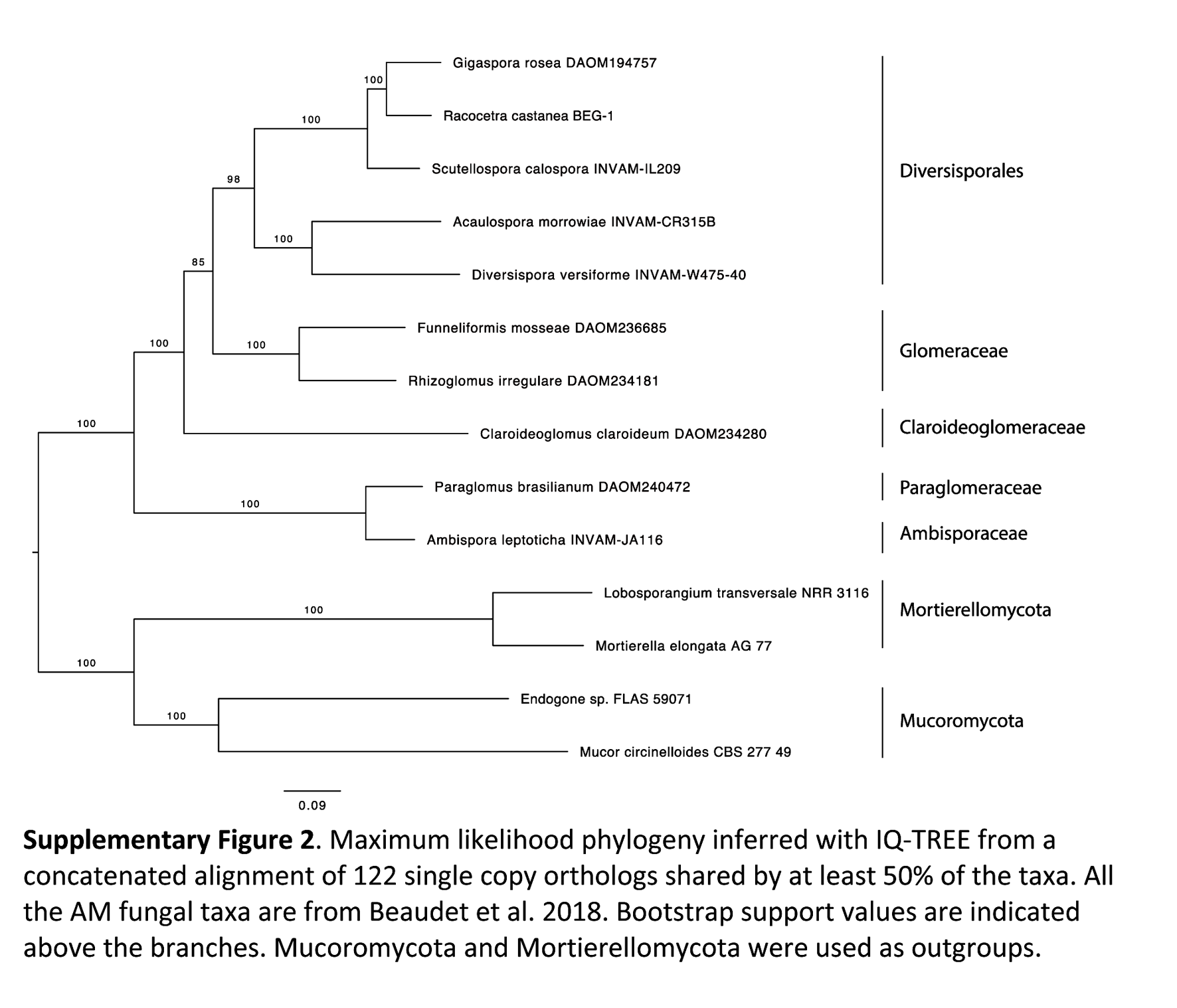

Supplement: Supplementary file 2 [file Image_2.TIF]

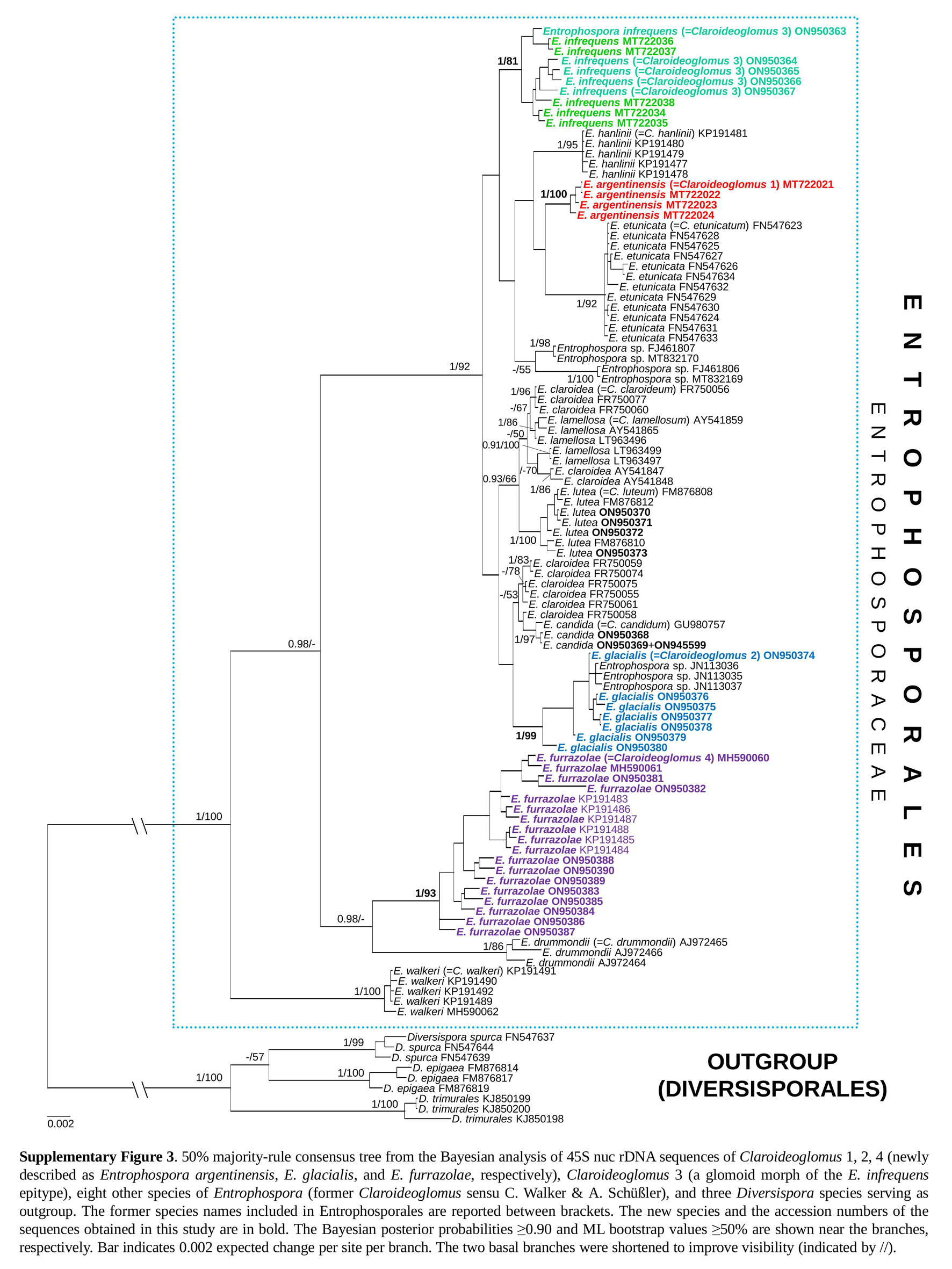

Supplement: Supplementary file 3 [file Image_3.JPEG]

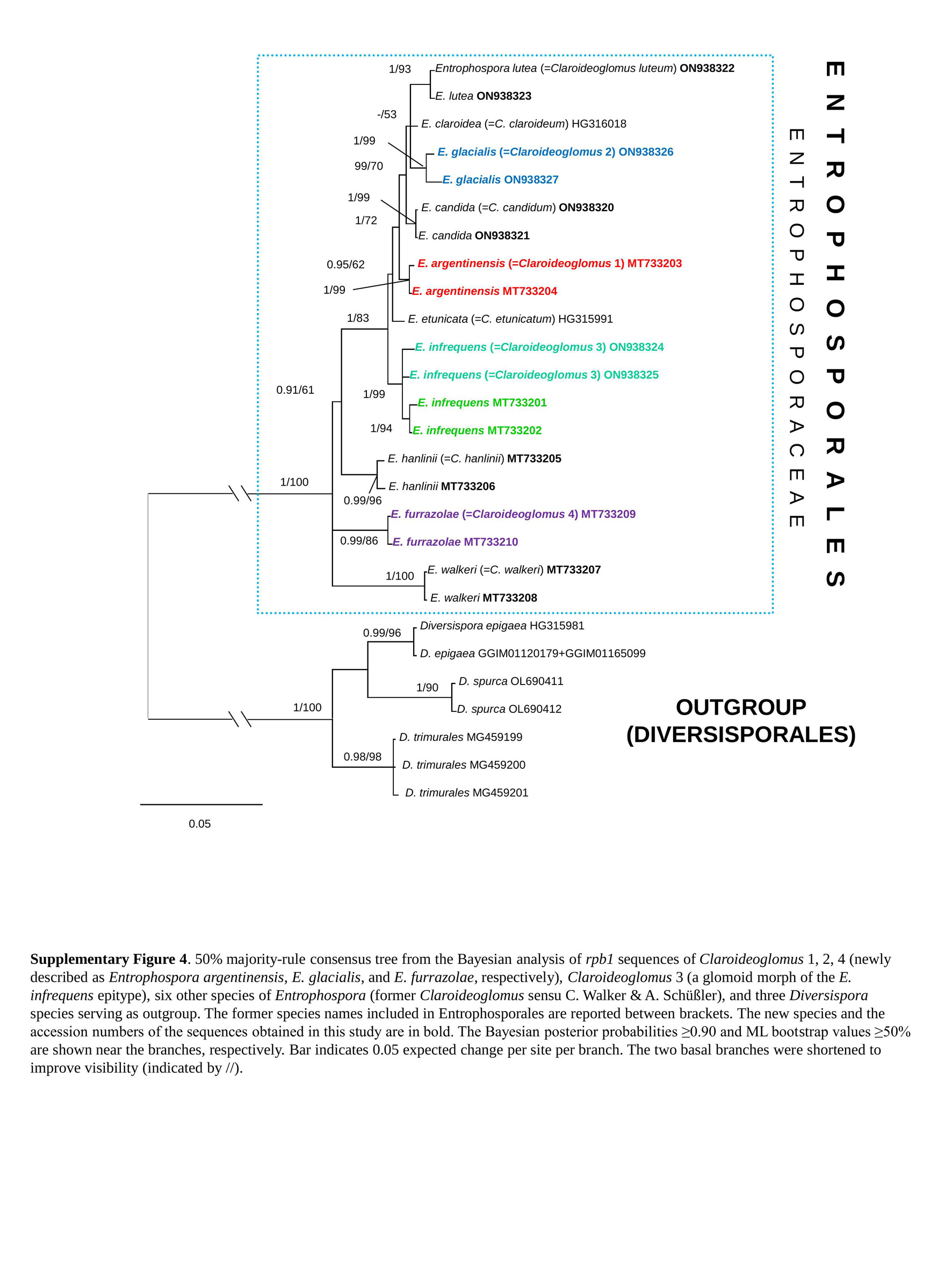

Supplement: Supplementary file 4 [file Image_4.JPEG]

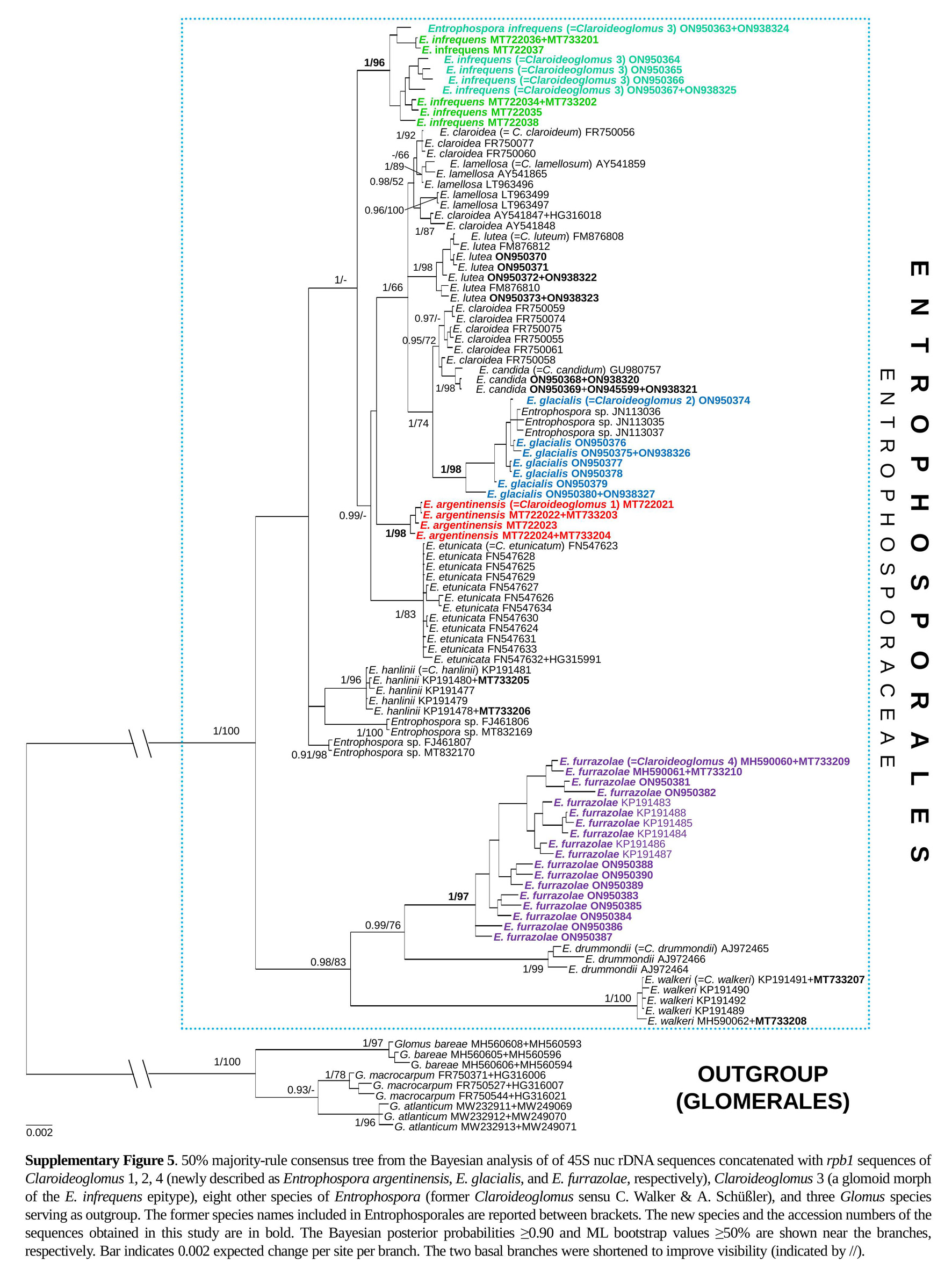

Supplement: Supplementary file 5 [file Image_5.JPEG]
