# Supplementary material for: A new order, Entrophosporales, and three new Entrophospora species in Glomeromycota
Source: Front Microbiol. 2022 Nov 29;13:962856. doi: 10.3389/fmicb.2022.962856 (PMC9835108; doi:10.3389/fmicb.2022.962856)
Supplement: Supplementary file 6 [file Table_1.DOCX]

**Supplementary Table 1**. Taxa included in the phylogenomic analyses.

| **Species** | **Isolate** | **Publication** |
| --- | --- | --- |
| **Data included in Montoliu-Nerin et al. 2021** | | |
| *Acaulospora colombiana* | CL356 | Montoliu-Nerin *et al.*, 2021 |
| *Acaulospora morrowiae* | CL551 | Montoliu-Nerin *et al.* 2021 |
| *Ambispora gerdemannii* | MT106 | Montoliu-Nerin *et al.* 2021 |
| *Ambispora leptoticha* | FL130A | Montoliu-Nerin *et al.* 2021 |
| *Claroideoglomus luteum* | SA 101 | Montoliu-Nerin *et al.*, 2020 |
| *Claroideoglomus candidum* | NC172 | Montoliu-Nerin *et al.* 2021 |
| *Claroideoglomus candidum* | CCK pot B 6-9 | Montoliu-Nerin *et al.* 2021 |
| *Cetraspora pellucida* | FL966 | Montoliu-Nerin *et al.* 2021 |
| *Cetraspora pellucida* | 28Kansas | Montoliu-Nerin *et al.* 2021 |
| *Dentiscutata erythropus* | MA453B | Montoliu-Nerin *et al.* 2021 |
| *Dentiscutata heterogama* | IL203A | Montoliu-Nerin *et al.* 2021 |
| *Diversispora eburnea* | AZ414A | Montoliu-Nerin *et al.* 2021 |
| *Diversispora epigaea* | IT104 | Sun *et al.*, 2018 |
| *Funneliformis caledonius* | UK204 | Montoliu-Nerin *et al.* 2021 |
| *Funneliformis mosseae* | 87-6 pot B 2015 | Montoliu-Nerin *et al.* 2021 |
| *Gigaspora margarita* | 120-4 pot B 10/14 | Montoliu-Nerin *et al.* 2021 |
| *Gigaspora rosea* | FL105 | Montoliu-Nerin *et al.* 2021 |
| *Oehlia diaphana* | DAOM227022 | Morin *et al.*, 2019 |
| *Paraglomus brasilianum* | BR232B | Montoliu-Nerin *et al.* 2021 |
| *Paraglomus occultum* | IA702 | Montoliu-Nerin *et al.* 2021 |
| *Racocetra fulgida* | IN212 | Montoliu-Nerin *et al.* 2021 |
| *Racocetra persica* | MA461A | Montoliu-Nerin *et al.* 2021 |
| *Rhizoglomus irregulare* | DAOM197198 | Montoliu-Nerin *et al.* 2021 |
| *Rhizoglomus irregulare* | A1 | Chen *et al.*, 2018 |
| *Rhizoglomus irregulare* | MUCL43196 | Morin *et al.*, 2019 |
| *Scutellospora calospora* | AU212A | Montoliu-Nerin *et al.* 2021 |
| **Data included in Beaudet et al. 2018** | | |
| *Acaulospora morrowiae* | CR315B | Beaudet *et al.*, 2018 |
| *Ambispora lepototicha* | JA116 | Beaudet *et al.*, 2018 |
| *Claroideoglomus claroideum* | DAOM234280 | Beaudet *et al.*, 2018 |
| *Diversispora versiforme* | W475 40 | Beaudet *et al.*, 2018 |
| *Funneliformis mosseae* | DAOM236685 | Beaudet *et al.*, 2018 |
| *Gigaspora rosea* | DAOM194757 | (Tang et al., 2016) |
| *Paraglomus brasilianum* | DAOM240472 | Beaudet *et al.*, 2018 |
| *Racocetra castanea* | BEG 1 | Beaudet *et al.*, 2018 |
| *Rhizophagus irregularis* | DAOM234181 | Beaudet *et al.*, 2018 |
| *Scutellospora calospora* | IL209 | Beaudet *et al.*, 2018 |
| **Outgroups** | | |
| *Mortierella elongata* | AG 77 | Uehling *et al.*, 2017 |
| *Lobosporangium transversale* | NRR 3116 | Mondo *et al.*, 2017 |
| *Endogone sp.* | FLAS 59071 | Chang *et al.*, 2019 |
| *Mucor circinelloides* | CBS 277 49 | Corrochano *et al.*, 2016 |

**References**

Beaudet, D., Chen, E.C.H., Mathieu, S., Yildirir, G., Ndikumana, S., Dalpé, Y., Séguin, S., Farinelli, L., Stajich, J.E., Corradi, N., 2018. Ultra-low input transcriptomics reveal the spore functional content and phylogenetic affiliations of poorly studied arbuscular mycorrhizal fungi. DNA Res. https://doi.org/10.1093/dnares/dsx051

Chang, Y., Desirò, A., Na, H., Sandor, L., Lipzen, A., Clum, A., Barry, K., Grigoriev, I. V., Martin, F.M., Stajich, J.E., Smith, M.E., Bonito, G., Spatafora, J.W., 2019. Phylogenomics of Endogonaceae and evolution of mycorrhizas within Mucoromycota. New Phytol. https://doi.org/10.1111/nph.15613

Chen, E.C.H., Morin, E., Beaudet, D., Noel, J., Yildirir, G., Ndikumana, S., Charron, P., St-Onge, C., Giorgi, J., Krüger, M., Marton, T., Ropars, J., Grigoriev, I. V., Hainaut, M., Henrissat, B., Roux, C., Martin, F., Corradi, N., 2018. High intraspecific genome diversity in the model arbuscular mycorrhizal symbiont *Rhizophagus irregularis*. New Phytol. https://doi.org/10.1111/nph.14989

Corrochano, L.M., Kuo, A., Marcet-Houben, M., Polaino, S., Salamov, A., Villalobos-Escobedo, J.M., Grimwood, J., Álvarez, M.I., Avalos, J., Bauer, D., Benito, E.P., Benoit, I., Burger, G., Camino, L.P., Cánovas, D., Cerdá-Olmedo, E., Cheng, J.F., Domínguez, A., Eliáš, M., Eslava, A.P., Glaser, F., Gutiérrez, G., Heitman, J., Henrissat, B., Iturriaga, E.A., Lang, B.F., Lavín, J.L., Lee, S.C., Li, W., Lindquist, E., López-García, S., Luque, E.M., Marcos, A.T., Martin, J., McCluskey, K., Medina, H.R., Miralles-Durán, A., Miyazaki, A., Muñoz-Torres, E., Oguiza, J.A., Ohm, R.A., Olmedo, M., Orejas, M., Ortiz-Castellanos, L., Pisabarro, A.G., Rodríguez-Romero, J., Ruiz-Herrera, J., Ruiz-Vázquez, R., Sanz, C., Schackwitz, W., Shahriari, M., Shelest, E., Silva-Franco, F., Soanes, D., Syed, K., Tagua, V.G., Talbot, N.J., Thon, M.R., Tice, H., de Vries, R.P., Wiebenga, A., Yadav, J.S., Braun, E.L., Baker, S.E., Garre, V., Schmutz, J., Horwitz, B.A., Torres-Martínez, S., Idnurm, A., Herrera-Estrella, A., Gabaldón, T., Grigoriev, I. V., 2016. Expansion of Signal Transduction Pathways in Fungi by Extensive Genome Duplication. Curr. Biol. https://doi.org/10.1016/j.cub.2016.04.038

Mondo, S.J., Dannebaum, R.O., Kuo, R.C., Louie, K.B., Bewick, A.J., LaButti, K., Haridas, S., Kuo, A., Salamov, A., Ahrendt, S.R., Lau, R., Bowen, B.P., Lipzen, A., Sullivan, W., Andreopoulos, B.B., Clum, A., Lindquist, E., Daum, C., Northen, T.R., Kunde-Ramamoorthy, G., Schmitz, R.J., Gryganskyi, A., Culley, D., Magnuson, J., James, T.Y., O’Malley, M.A., Stajich, J.E., Spatafora, J.W., Visel, A., Grigoriev, I. V., 2017. Widespread adenine N6-methylation of active genes in fungi. Nat. Genet. https://doi.org/10.1038/ng.3859

Montoliu-Nerin, M., Sánchez-García, M., Bergin, C., Grabherr, M., Ellis, B., Kutschera, V.E., Kierczak, M., Johannesson, H., Rosling, A., 2020. Building de novo reference genome assemblies of complex eukaryotic microorganisms from single nuclei. Sci. Rep. 10. https://doi.org/10.1038/s41598-020-58025-3

Montoliu-Nerin, M., Sánchez-García, M., Bergin, C., Kutschera, V.E., Johannesson, H., Bever, J.D., Rosling, A., 2021. In-depth Phylogenomic Analysis of Arbuscular Mycorrhizal Fungi Based on a Comprehensive Set of de novo Genome Assemblies . Front. Fungal Biol. .

Morin, E., Miyauchi, S., San Clemente, H., Chen, E.C.H., Pelin, A., de la Providencia, I., Ndikumana, S., Beaudet, D., Hainaut, M., Drula, E., Kuo, A., Tang, N., Roy, S., Viala, J., Henrissat, B., Grigoriev, I. V., Corradi, N., Roux, C., Martin, F.M., 2019. Comparative genomics of Rhizophagus irregularis, R. cerebriforme, R. diaphanus and Gigaspora rosea highlights specific genetic features in Glomeromycotina. New Phytol. https://doi.org/10.1111/nph.15687

Sun, X., Chen, W., Ivanov, S., MacLean, A.M., Wight, H., Ramaraj, T., Mudge, J., Harrison, M.J., Fei, Z., 2018. Genome and evolution of the arbuscular mycorrhizal fungus Diversispora epigaea (formerly Glomus versiforme) and its bacterial endosymbionts. New Phytol. 0. https://doi.org/10.1111/nph.15472

Tang, N., San Clemente, H., Roy, S., Bécard, G., Zhao, B., Roux, C., 2016. A Survey of the Gene Repertoire of Gigaspora rosea Unravels Conserved Features among Glomeromycota for Obligate Biotrophy. Front. Microbiol. 7, 233. https://doi.org/10.3389/fmicb.2016.00233

Uehling, J., Gryganskyi, A., Hameed, K., Tschaplinski, T., Misztal, P.K., Wu, S., Desirò, A., Vande Pol, N., Du, Z., Zienkiewicz, A., Zienkiewicz, K., Morin, E., Tisserant, E., Splivallo, R., Hainaut, M., Henrissat, B., Ohm, R., Kuo, A., Yan, J., Lipzen, A., Nolan, M., LaButti, K., Barry, K., Goldstein, A.H., Labbé, J., Schadt, C., Tuskan, G., Grigoriev, I., Martin, F., Vilgalys, R., Bonito, G., 2017. Comparative genomics of Mortierella elongata and its bacterial endosymbiont Mycoavidus cysteinexigens. Environ. Microbiol. https://doi.org/10.1111/1462-2920.13669
