# Supplementary material for: A new order, Entrophosporales, and three new Entrophospora species in Glomeromycota
Source: Front Microbiol. 2022 Nov 29;13:962856. doi: 10.3389/fmicb.2022.962856 (PMC9835108; doi:10.3389/fmicb.2022.962856)
Supplement: Supplementary file 7 [file Table_2.DOCX]

**Supplementary Table 2.** Number of AMF species analyzed, BI and ML support values, and resolution of phylogenetic trees generated from analyses of single locus and concatenated loci.

| Locus | No. of species | No. of species clades supported  with BI ≥0.95/ ML ≥70%* | | Mean BI and ML values for supported species clades | | Mean supports of nodes with BI ≥0.95/ML ≥70%** | | Resolution*** | |
| --- | --- | --- | --- | --- | --- | --- | --- | --- | --- |
|  |  | BI | ML | BI | ML | BI | ML | BI | ML |
| 45S | 14 | 12 | 12 | 1 | 95.1 | 0.99 | 87.7 | 0.62 | 0.49 |
| *rpb1* | 10 | 8 | 8 | 1 | 96.4 | 0.99 | 89.5 | 0.87 | 0.72 |
| 45S+*rpb1* | 14 | 12 | 12 | 0.99 | 96.8 | 0.99 | 87.9 | 0.69 | 0.52 |
| 45S+*rpb1*_G | 14 | 11 | 12 | 0.99 | 96.8 | 0.99 | 87.9 | 0.68 | 0.54 |

* branches and supports of nodes binding branches with species provided with single sequences were not considered in calculations.

** the sums of BI ≥0.95 and ML ≥70% supports divided by the number of BI ≥0.95 and ML ≥70% supports.

*** the maximum number of significantly supported internal branches of the ingroup divided by the size of the ingroup (*n-*1).
