# Supplementary material for: A new order, Entrophosporales, and three new Entrophospora species in Glomeromycota
Source: Front Microbiol. 2022 Nov 29;13:962856. doi: 10.3389/fmicb.2022.962856 (PMC9835108; doi:10.3389/fmicb.2022.962856)
Supplement: Supplementary file 8 [file Table_3.DOCX]

**Supplementary Table 3.** The results of EPA affiliation of the sequences selected querying the GenBank database with rDNA sequences of the new species. Each selected sequence is defined by its accession number (first column), the accumulated likelihood weights assigned to the taxonomic path (second column), the taxon assigned in the reference tree (third column), and the geographic information of the source, when available (fourth column).

name aLWR affiliation

KX643976 0.9946 Entrophospora_infrequens France, Mas d'Imbert

KX643934 0.9886 Entrophospora_infrequens France, Mas d'Imbert

FR871351 0.995 Entrophospora_infrequens Spain, Murcia

FR871314 0.995 Entrophospora_infrequens Spain, Murcia

FR871350 1 Entrophospora_infrequens Spain, Murcia

FR871352 0.9786 Entrophospora_infrequens Spain, Murcia

FR871315 0.9721 Entrophospora_infrequens Spain, Murcia

MT722039 1 Entrophospora_infrequens Poland

HF970233 0.9996 Entrophospora_infrequens Peru, Junin

HE775325 0.9977 Entrophospora_infrequens Czech Republic, North Bohemia, Malesov

JN937203 1 Entrophospora_infrequens USA, New York State

JN937224 0.9871 Entrophospora_infrequens USA, New York State

JN937290 0.9871 Entrophospora_infrequens USA, New York State

JN937294 0.994 Entrophospora_infrequens USA, New York State

JN937248 0.9859 Entrophospora_infrequens USA, New York State

JN937315 1 Entrophospora_infrequens USA, New York State

JN937251 1 Entrophospora_infrequens USA, New York State

JN937257 1 Entrophospora_infrequens USA, New York State

JN937227 1 Entrophospora_infrequens USA, New York State

JN937274 1 Entrophospora_infrequens USA, New York State

JN937306 0.9838 Entrophospora_infrequens USA, New York State

JN937268 0.9654 Entrophospora_infrequens USA, New York State

JN937281 1 Entrophospora_infrequens USA, New York State

JN937301 1 Entrophospora_infrequens USA, New York State

JN937318 1 Entrophospora_infrequens USA, New York State

JN937308 0.9099 Entrophospora_infrequens USA, New York State

JN937536 1 Entrophospora_infrequens USA, New York State

JN937242 0.9719 Entrophospora_infrequens USA, New York State

AY639341 1 Entrophospora_argentinensis Switzerland

AY639343 1 Entrophospora_argentinensis Switzerland

AY639333 1 Entrophospora_argentinensis Switzerland

AY639339 1 Entrophospora_argentinensis Switzerland

AY639346 1 Entrophospora_argentinensis Switzerland

AY639347 1 Entrophospora_argentinensis Switzerland

AY639334 1 Entrophospora_argentinensis Switzerland

AY639337 1 Entrophospora_argentinensis Switzerland

JF342363 0.9954 Entrophospora_glacialis Switzerland

HQ117875 0.9925 Entrophospora_glacialis Brazil

JN937162 1 Entrophospora_glacialis USA, New York State

LT964731 1 Entrophospora_glacialis Norway, Buskerud, lake Mjosa

LT964732 1 Entrophospora_glacialis Norway, Buskerud, lake Mjosa

LT964733 1 Entrophospora_glacialis Norway, Buskerud, lake Mjosa

AY639342 1 Entrophospora_glacialis Switzerland

AY639331 1 Entrophospora_glacialis Switzerland

AY639344 1 Entrophospora_glacialis Switzerland

AY639335 1 Entrophospora_glacialis Switzerland

AY639345 1 Entrophospora_glacialis Switzerland

AY639332 1 Entrophospora_glacialis Switzerland

AB665520 1 Entrophospora_glacialis Japan, Hokkaido, Sapporo

AB561128 1 Entrophospora_glacialis Japan, Hokkaido, Ishikari

AB369773 1 Entrophospora_glacialis Japan, Okinawa, Nago

AB547182 0.971 Entrophospora_glacialis Japan, Hokkaido, Sapporo

GU322903 1 Entrophospora_glacialis Netherlands

JQ218224 0.9831 Entrophospora_glacialis China

AJ271927 1 Entrophospora_glacialis Poland

AM040315 1 Entrophospora_glacialis Japan, Niigata

AM040314 1 Entrophospora_glacialis Japan, Niigata

JN937145 1 Entrophospora_argentinensis USA, New York State

HQ857082 1 Entrophospora_glacialis Switzerland

HQ857062 1 Entrophospora_glacialis Switzerland

HQ857083 1 Entrophospora_glacialis Switzerland

HQ857088 1 Entrophospora_glacialis Switzerland

HQ857095 1 Entrophospora_glacialis Switzerland

AY639193 0.9845 Entrophospora_glacialis Switzerland

AY639194 0.9811 Entrophospora_glacialis Switzerland

AY639192 0.9809 Entrophospora_glacialis Switzerland

HQ856937 0.976 Entrophospora_infrequens Brazil

AM040312 0.9932 Entrophospora_glacialis Japan, Niigata

AM040330 0.9943 Entrophospora_glacialis Japan, Niigata

ON033161 1 Entrophospora_furrazolae Switzerland

ON033158 1 Entrophospora_furrazolae

ON033159 0.9964 Entrophospora_furrazolae

ON033160 0.9958 Entrophospora_furrazolae

MK275297 0.9916 Entrophospora_furrazolae

ON033156 0.9996 Entrophospora_furrazolae

MK275296 0.9985 Entrophospora_furrazolae

ON033155 0.9992 Entrophospora_furrazolae

ON033154 0.9993 Entrophospora_furrazolae

MK275298 0.9972 Entrophospora_furrazolae

ON033162 0.9863 Entrophospora_furrazolae

ON033157 0.992 Entrophospora_furrazolae

ON033153 0.9898 Entrophospora_furrazolae

ON033152 0.9894 Entrophospora_furrazolae

FR871354 0.9932 Entrophospora_furrazolae Spain

FR871353 0.9958 Entrophospora_furrazolae

FR871390 0.9937 Entrophospora_furrazolae Spain

JX096582 1 Entrophospora_furrazolae China

JQ218217 1 Entrophospora_furrazolae China

HG425962 1 Entrophospora_furrazolae Czech Republic, Central Bohemia, Melnik

HG425961 0.9995 Entrophospora_furrazolae Czech Republic, Central Bohemia, Melnik

HG425960 1 Entrophospora_furrazolae Czech Republic, Central Bohemia, Melnik

MK275293 1 Entrophospora_furrazolae

MK275294 1 Entrophospora_furrazolae
